# Supplementary material for: Engineering an Escherichia coli strain for production of long single-stranded DNA
Source: Nucleic Acids Res. 2024 Mar 18;52(7):4098–107. doi: 10.1093/nar/gkae189 (PMC11040142; doi:10.1093/nar/gkae189)
Supplement: gkae189_Supplemental_File [file gkae189_supplemental_file.docx]

# **Supplementary Figures**


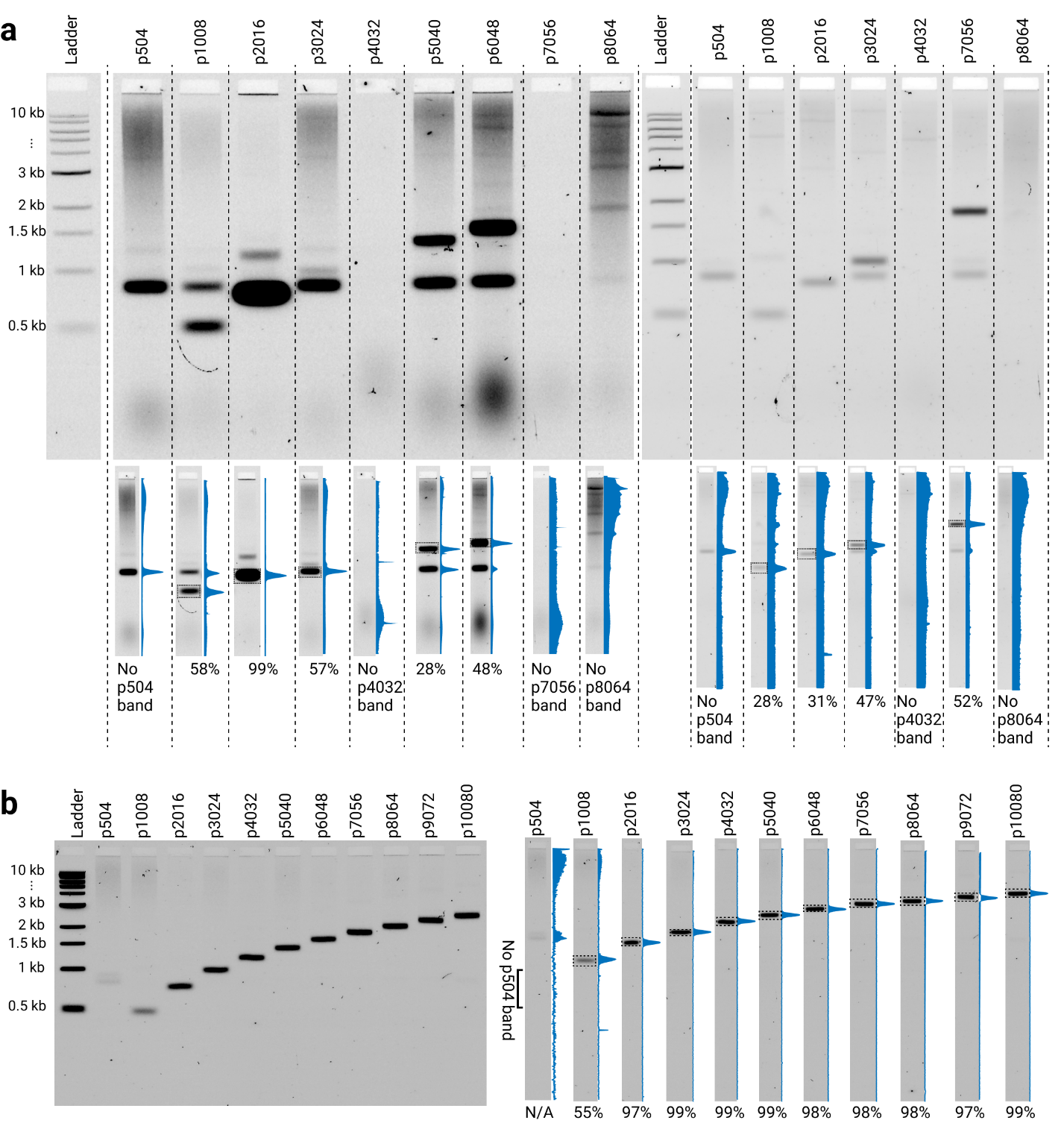


**Fig. S1** **ssDNA products generated by helper plasmids are of inconsistent quality**. **a)** Agarose gels showing ssDNA products produced by TG1 bacteria dual-transformed with both M13mp18 helper plasmids and pScaf-based phagemids (left) show inconsistent products. Except for the 2016-nt ssDNA sample, the majority band of all lanes composed of less than 60% of the lane intensity. **b)** Agarose gel from ssDNA produced by eScaf. For ssDNA products 2016-nt and longer, the purity was consistently greater than 95%. All ladders are 1 kb ladders from New England Biolabs. Reported yields were calculated by integrating the intensity of the target band and dividing by the integrated intensities of all features.


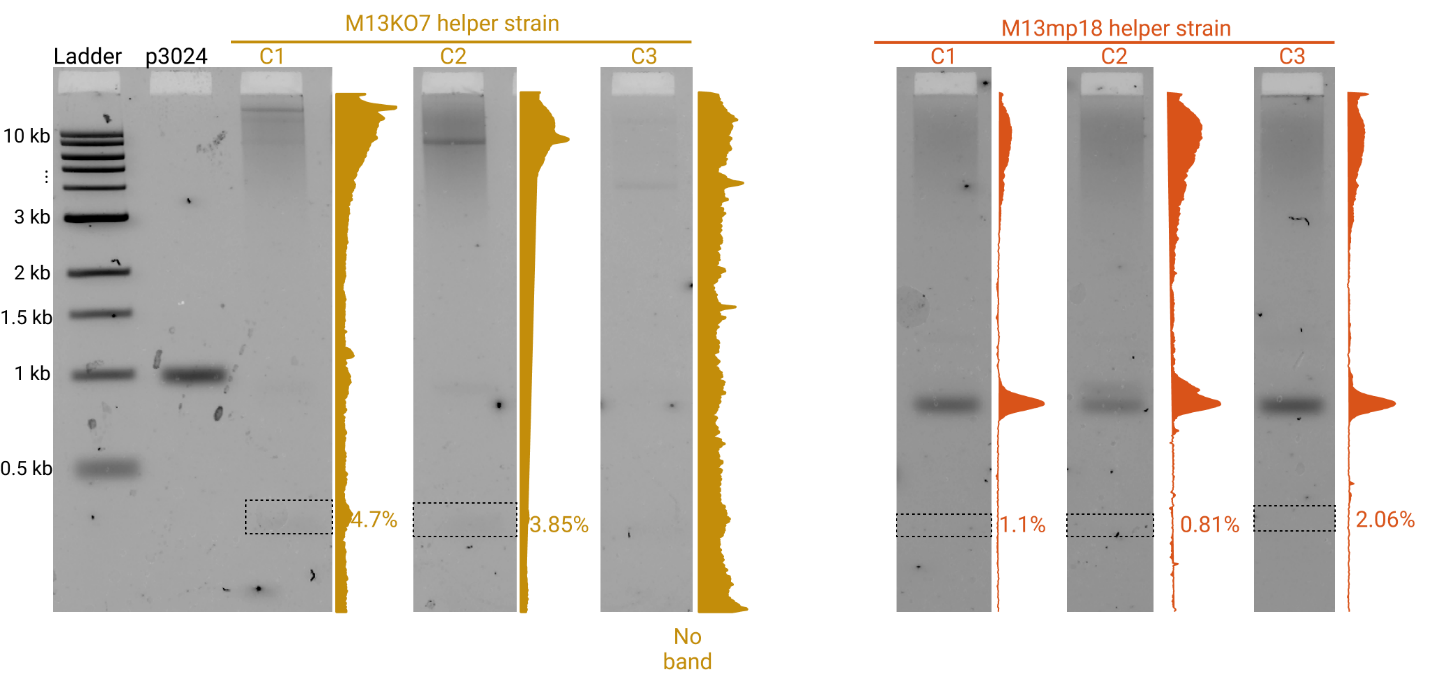


**Fig. S2**. **Short ssDNA is not well produced by either the M13KO7 or M13mp18 helper strains.** Quantification of 504-nt ssDNA band in M13KO7 helper strains show slightly higher products than in M13mp18 helper strains, but in both strains, 504-nt ssDNA production is poor. Ladder is a 1 kb ladder from New England Biolabs.


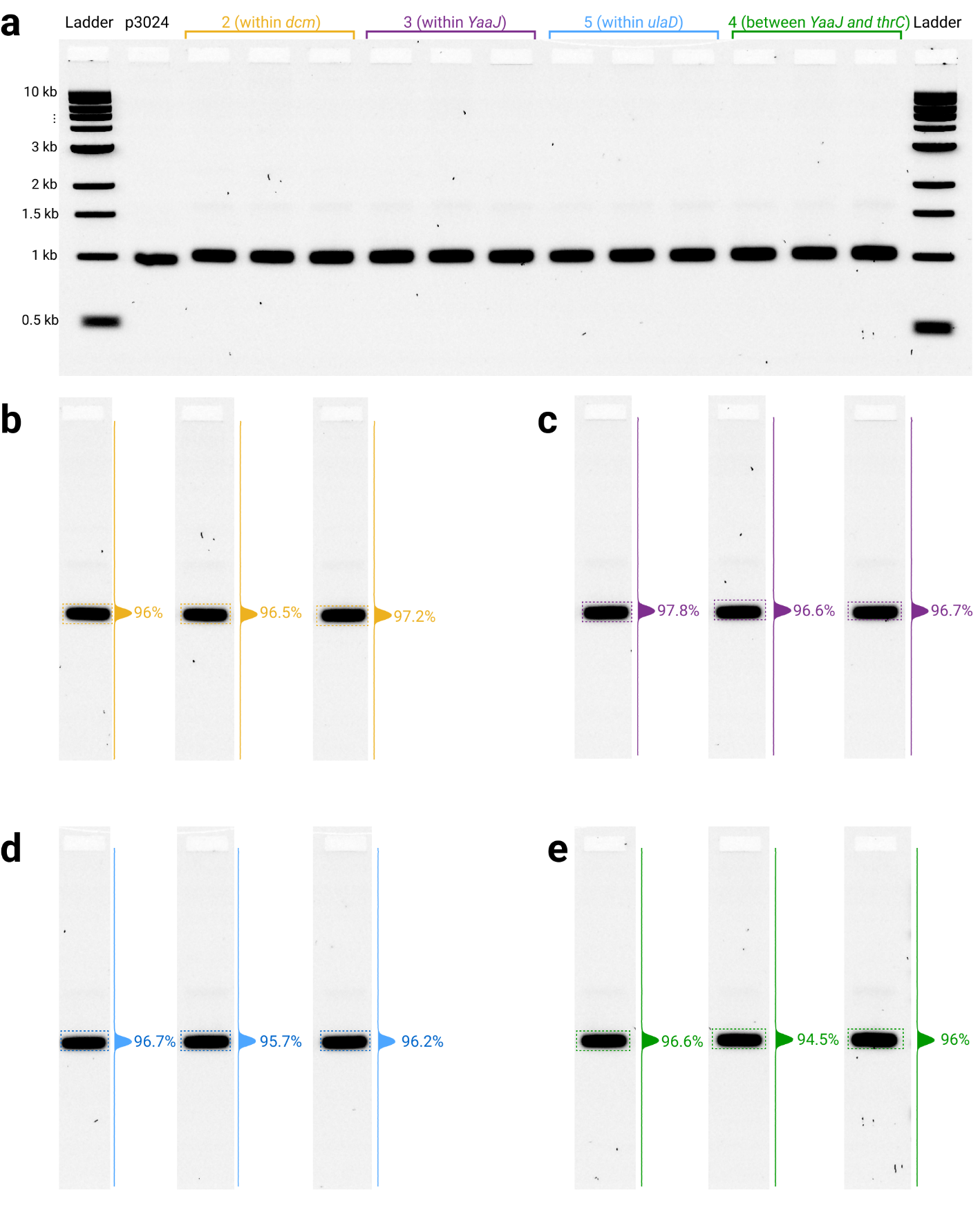


**Fig. S3 Scaffold purity is not affected by integration location. a)** Full agarose gel showing species within ssDNA products integrated into four different locations. Target is a 3024-nt ssDNA product. Ladders are 1 kb ladders from New England Biolabs. **b,c,d,e)** 1D line histograms showing majority of signal (between 95~97%) is located in the target band for all samples over all integration locations.


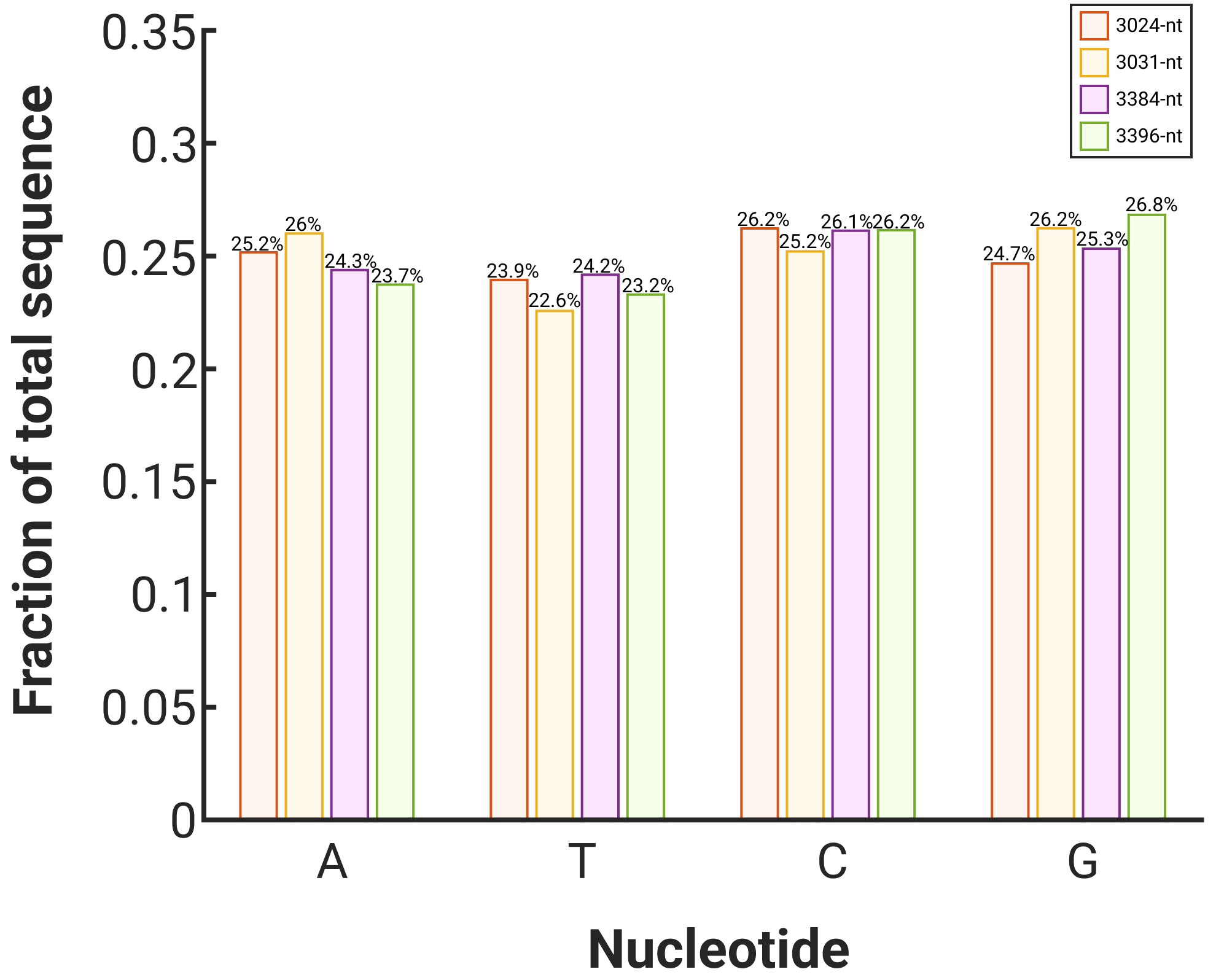


**Fig. S4** **Nucleotide composition in ~3000-nt ssDNA products are all similar.** Base composition ranges between 22% and 27%. No nucleotide composition varies more than 3% between any ssDNA product, suggesting that differences in ssDNA titer are not due to composition alone.

**
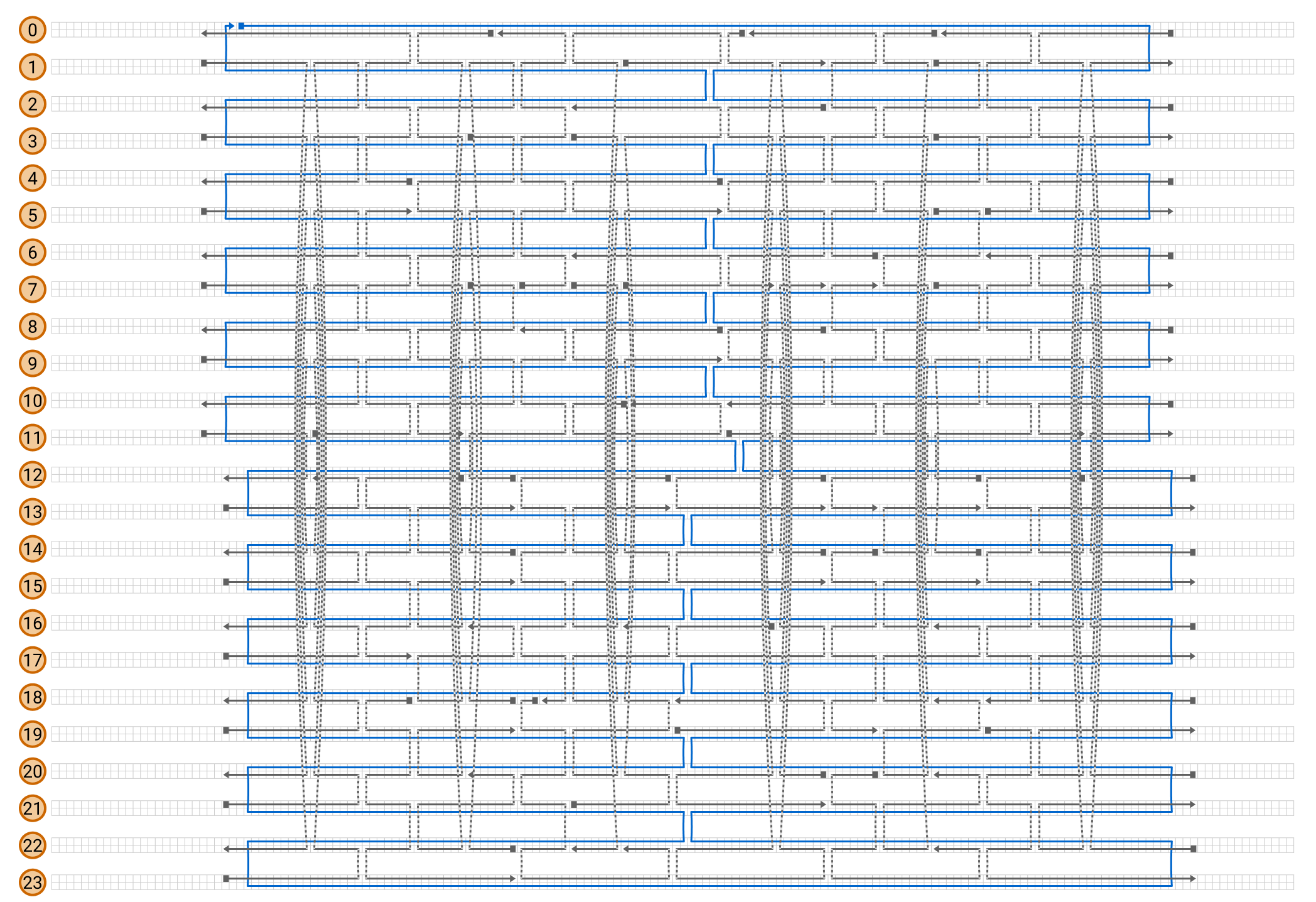
**

**Fig. S5 Cadnano strand diagram of DNA origami tile**. The custom 3024-nt scaffold strand is represented by the blue line. The staples are represented by grey lines. Dotted grey lines represent staple crossovers.


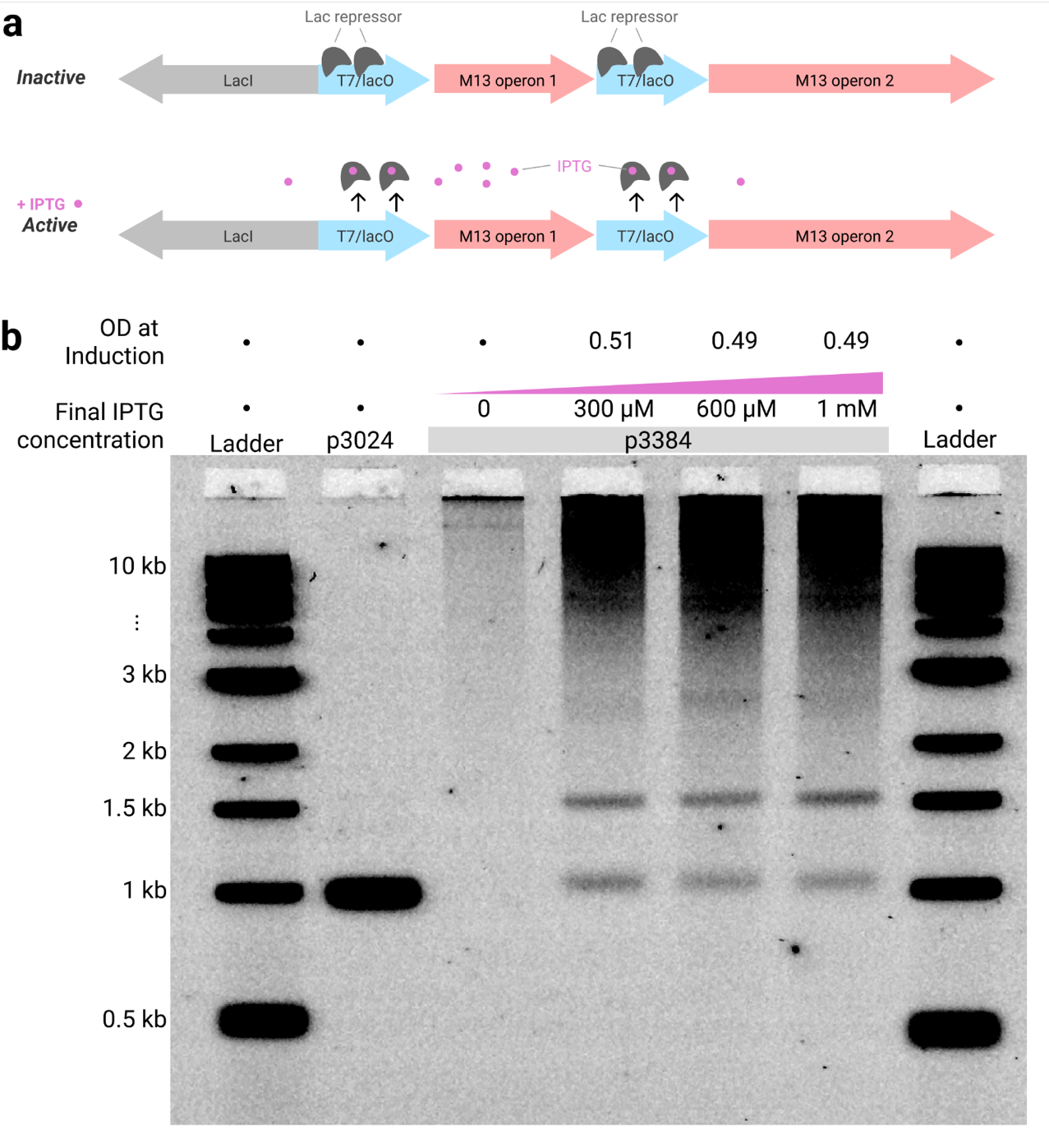


**Fig. S6** **T7-regulated M13mp18-helper strains show inducible production of ssDNA, but ssDNA products are impure and have high background**. **a)** Schematic of T7-regulated M13mp18 cassette. We replaced two native promoters in the M13mp18 genome with T7lac and placed the *LacI* gene upstream of the c­­assette. We integrated this entire cassette into the NEB T7express *E. coli* genome. **b)** We transformed our T7-regulated helper with the p3384 pScaf phagemid. Liquid cultures were grown at 30 °C and when the O.D.600 reached 0.5, induced the cultures at varying levels of IPTG. ssDNA products were collected 3-4 hours post induction and run on a gel alongside a 1 kb ladder from New England Biolabs. Target band (near 1 kb band) appears when induced, but significant off-target species (large smear near well, higher order bands near 1.5 kb band in ladder) also appear.

**DNA sequences used in this work**

| **Description** | **Sequence** |
| --- | --- |
| 504-nt ssDNA sequence for length test | aatagtggactcttgttccaaactggaacaacactcaaccctatctcgggctattcttttgatttataagggattttgccgatttcggaacgggtacctacgaagagttccagcagggattccaagaaatggccaatgaagattgctgagagcagcactgtgcaagactggaaaggtggtgaagcaatacaccatgaatcccggatcgctgacgcgccctgtagcggcgcattaagcgcggcgggtgtggtggttacgcgcagcgtgaccgctacacttgccagcgccctagcgcccgctcctttcgctttcttcccttcctttctcgccacgttcgccggctttccccgtcaagctctaaatcgggggctccctttagggttccgatttagtgctttacggcacctcgaccccaaaaaacttgatttgggtgatggttcacgtagtgggccatcgccctgatagacggtttttcgccctttgacgttggagtccacgttcttt |
| 1008-nt ssDNA sequence for length test | aatagtggactcttgttccaaactggaacaacactcaaccctatctcgggctattcttttgatttataagggattttgccgatttcggaacgggtacctacgaagagttccagcagggattccaagaaatggccaatgaagattgtttcccgcacctgggctctttcgcgccaatcgacgttaaacacttgaatttaagatgagcatcgcagaagtggatgatatgcgcgcctacggtccaatcggctgggtgagcgtccctataatctcacggataccgacgaaagtagggcaataatcgcgctgcatgagaacggatagcccggatgcaaaggtggaacgtaattgttaagagaagcaaatacgttaagctatcatttcccgtaaatcttttacatacgtaaggtggaagtctaaactagcctagctgcacaagcatcggactgcttgccctctatctttgtggaagttcaaggtaatggaagcggtagaacgtatgatcgtcacgcagacctaaagaatcacgccgcgcttacgtccaaccttgggacctacatctgtacagacccaggtccaggccgaggttatggatgtagatttgacttggctgatctacagtctcgagttacaatggcgtcactgctacctgctgagagcagcactgtgcaagactggaaaggtggtgaagcaatacaccatgaatcccggatcgctgacgcgccctgtagcggcgcattaagcgcggcgggtgtggtggttacgcgcagcgtgaccgctacacttgccagcgccctagcgcccgctcctttcgctttcttcccttcctttctcgccacgttcgccggctttccccgtcaagctctaaatcgggggctccctttagggttccgatttagtgctttacggcacctcgaccccaaaaaacttgatttgggtgatggttcacgtagtgggccatcgccctgatagacggtttttcgccctttgacgttggagtccacgttcttt |
| 2016-nt ssDNA sequence for length test | aatagtggactcttgttccaaactggaacaacactcaaccctatctcgggctattcttttgatttataagggattttgccgatttcggaacgggtacctacgaagagttccagcagggattccaagaaatggccaatgaagattgtttcccgcacctgggctctttcgcgccaatcgacgttaaacacttgaatttaagatgagcatcgcagaagtggatgatatgcgcgcctacggtccaatcggctgggtgagcgtccctataatctcacggataccgacgaaagtagggcaataatcgcgctgcatgagaacggatagcccggatgcaaaggtggaacgtaattgttaagagaagcaaatacgttaagctatcatttcccgtaaatcttttacatacgtaaggtggaagtctaaactagcctagctgcacaagcatcggactgcttgccctctatctttgtggaagttcaaggtaatggaagcggtagaacgtatgatcgtcacgcagacctaaagaatcacgccgcgcttacgtccaaccttgggacctacatctgtacagacccaggtccaggccgaggttatggatgtagatttgacttggctgatctacagtctcgagttacaatggcgtcactgctacctccatatgaaggataagaggcagccacaactcagccaccatcctaccggaaacgctttaagggcgaacgtgaagggaagtgcctacacgaccctctatgtgggaagtagtctacgatacacgatgattggaagtgtggcggcataggatgcatctacggaccgacactagttatagaagggtgcagtccttgacgcatacgaatagcgttagctcgcgatttcccgctgggttactcatggagagcggctcagacgcctagcccatactgaccgtgtaccaacgatccaccaattattttcagaacagcccttttatggcgaagggaaaacagagccttagtatgttacagtccgctctattgggagatctcgggcaggcggaagatattaattagaaccgcagaccgcatgatgtgccgtcctcaaatggcctggatatcccgtcacacgaattatctcagttgaagtgatcctttagggcccgagagcaccacaagctcccaaccgctgcgaagagtgctaacgagttgtcctatgttaagttattcgtatttaacccacgcccgcaccacctccgggaataatgtccaagatcatatcaaggaaacccagggcacgataagcggcacacagacaaccactttggctagttaagataacggatactctgacgtacagccggccattcgttctacttttgtgtagtcagatggtccgtaacaatggcccggctctgacaacaagtataagactgcatgacgggtgggtccacgttagatgttcgtgaggtagcccatacatcattctttacgaacaagcctctcccgagccagcagaggaggaatcagtgatcgttcacatggcgtacaacaaggcgtgcggcgccgtcggctagcgctgtgaacgtcgcagcgttgattccaaccctctgcccacgacttcgccgctaactggaagcggtattaaacccgaaggttccacgggtggccacttggtgaaggcggcatcgctgagagcagcactgtgcaagactggaaaggtggtgaagcaatacaccatgaatcccggatcgctgacgcgccctgtagcggcgcattaagcgcggcgggtgtggtggttacgcgcagcgtgaccgctacacttgccagcgccctagcgcccgctcctttcgctttcttcccttcctttctcgccacgttcgccggctttccccgtcaagctctaaatcgggggctccctttagggttccgatttagtgctttacggcacctcgaccccaaaaaacttgatttgggtgatggttcacgtagtgggccatcgccctgatagacggtttttcgccctttgacgttggagtccacgttcttt |
| 3024-nt ssDNA sequence for length test and composition test | aatagtggactcttgttccaaactggaacaacactcaaccctatctcgggctattcttttgatttataagggattttgccgatttcggaacgggtacctacgaagagttccagcagggattccaagaaatggccaatgaagattgtttcccgcacctgggctctttcgcgccaatcgacgttaaacacttgaatttaagatgagcatcgcagaagtggatgatatgcgcgcctacggtccaatcggctgggtgagcgtccctataatctcacggataccgacgaaagtagggcaataatcgcgctgcatgagaacggatagcccggatgcaaaggtggaacgtaattgttaagagaagcaaatacgttaagctatcatttcccgtaaatcttttacatacgtaaggtggaagtctaaactagcctagctgcacaagcatcggactgcttgccctctatctttgtggaagttcaaggtaatggaagcggtagaacgtatgatcgtcacgcagacctaaagaatcacgccgcgcttacgtccaaccttgggacctacatctgtacagacccaggtccaggccgaggttatggatgtagatttgacttggctgatctacagtctcgagttacaatggcgtcactgctacctccatatgaaggataagaggcagccacaactcagccaccatcctaccggaaacgctttaagggcgaacgtgaagggaagtgcctacacgaccctctatgtgggaagtagtctacgatacacgatgattggaagtgtggcggcataggatgcatctacggaccgacactagttatagaagggtgcagtccttgacgcatacgaatagcgttagctcgcgatttcccgctgggttactcatggagagcggctcagacgcctagcccatactgaccgtgtaccaacgatccaccaattattttcagaacagcccttttatggcgaagggaaaacagagccttagtatgttacagtccgctctattgggagatctcgggcaggcggaagatattaattagaaccgcagaccgcatgatgtgccgtcctcaaatggcctggatatcccgtcacacgaattatctcagttgaagtgatcctttagggcccgagagcaccacaagctcccaaccgctgcgaagagtgctaacgagttgtcctatgttaagttattcgtatttaacccacgcccgcaccacctccgggaataatgtccaagatcatatcaaggaaacccagggcacgataagcggcacacagacaaccactttggctagttaagataacggatactctgacgtacagccggccattcgttctacttttgtgtagtcagatggtccgtaacaatggcccggctctgacaacaagtataagactgcatgacgggtgggtccacgttagatgttcgtgaggtagcccatacatcattctttacgaacaagcctctcccgagccagcagaggaggaatcagtgatcgttcacatggcgtacaacaaggcgtgcggcgccgtcggctagcgctgtgaacgtcgcagcgttgattccaaccctctgcccacgacttcgccgctaactggaagcggtattaaacccgaaggttccacgggtggccacttggtgaaggcggcatcttaaagttctgacctcaattactatggtgccgtcatctaaccaaccattaagaagtagaattgtaaccggttatcggaatcagtgtcgacatactcaatgcccttaccctccgtaactctatgttttccgggttgttcatatcggacatgctatcgcttagacgactcgtctatatagggcgttagtatatcatgggcgtactatcccggtcaatcgtctacgtcgcaaatcacctccctttgggcaggagagcacttctacactgcctggcgctggcaccgcacaatcaataatgagattgtccggtgagacttacaatcccatcagcgagtctgcaatcccgctgtcacgataacatagtactcgccttctggcggttcctcagctatcctaacgtctatctaaactcaatgtggcgttatttggttcaagcaggcgggtcgtcaacgcgaaacgtaagtcccacacggccgtaacgcaatgtggcaattccagagttaccgatcgaggccaatggcccggccaagccgcaaccgcatcctccgccggtaaaatgactaaatgagtggaaccgtcgcgcttacgctctcggtgcccggaaaactctacagcatattgtctcattggtcctttggcgtatacagacacttagactaactctgactgtcgagtaaattccaatgaacgccagcactcagtacgcacggtgcgtccagggatggttacaaagctagagatgtatgtgctctcatcgaataacccgtagtatttggacctgaataccctggaatccgagggtgagaccattttacgttatcactgtgacatgctttttccggtcaatacacgggaaactacggacgctatggtcgggataaacgccgcatcatgtcaagctgaggccgctaaacaaagcatcaaggtagatttatttacaagcaaggccttgcgggcccagtccatgcgctgctgagattacagggattccgtgccagcccttcaacgtcgctgagagcagcactgtgcaagactggaaaggtggtgaagcaatacaccatgaatcccggatcgctgacgcgccctgtagcggcgcattaagcgcggcgggtgtggtggttacgcgcagcgtgaccgctacacttgccagcgccctagcgcccgctcctttcgctttcttcccttcctttctcgccacgttcgccggctttccccgtcaagctctaaatcgggggctccctttagggttccgatttagtgctttacggcacctcgaccccaaaaaacttgatttgggtgatggttcacgtagtgggccatcgccctgatagacggtttttcgccctttgacgttggagtccacgttcttt |
| 4032-nt ssDNA sequence for length test | aatagtggactcttgttccaaactggaacaacactcaaccctatctcgggctattcttttgatttataagggattttgccgatttcggaacgggtacctacgaagagttccagcagggattccaagaaatggccaatgaagattgtttcccgcacctgggctctttcgcgccaatcgacgttaaacacttgaatttaagatgagcatcgcagaagtggatgatatgcgcgcctacggtccaatcggctgggtgagcgtccctataatctcacggataccgacgaaagtagggcaataatcgcgctgcatgagaacggatagcccggatgcaaaggtggaacgtaattgttaagagaagcaaatacgttaagctatcatttcccgtaaatcttttacatacgtaaggtggaagtctaaactagcctagctgcacaagcatcggactgcttgccctctatctttgtggaagttcaaggtaatggaagcggtagaacgtatgatcgtcacgcagacctaaagaatcacgccgcgcttacgtccaaccttgggacctacatctgtacagacccaggtccaggccgaggttatggatgtagatttgacttggctgatctacagtctcgagttacaatggcgtcactgctacctccatatgaaggataagaggcagccacaactcagccaccatcctaccggaaacgctttaagggcgaacgtgaagggaagtgcctacacgaccctctatgtgggaagtagtctacgatacacgatgattggaagtgtggcggcataggatgcatctacggaccgacactagttatagaagggtgcagtccttgacgcatacgaatagcgttagctcgcgatttcccgctgggttactcatggagagcggctcagacgcctagcccatactgaccgtgtaccaacgatccaccaattattttcagaacagcccttttatggcgaagggaaaacagagccttagtatgttacagtccgctctattgggagatctcgggcaggcggaagatattaattagaaccgcagaccgcatgatgtgccgtcctcaaatggcctggatatcccgtcacacgaattatctcagttgaagtgatcctttagggcccgagagcaccacaagctcccaaccgctgcgaagagtgctaacgagttgtcctatgttaagttattcgtatttaacccacgcccgcaccacctccgggaataatgtccaagatcatatcaaggaaacccagggcacgataagcggcacacagacaaccactttggctagttaagataacggatactctgacgtacagccggccattcgttctacttttgtgtagtcagatggtccgtaacaatggcccggctctgacaacaagtataagactgcatgacgggtgggtccacgttagatgttcgtgaggtagcccatacatcattctttacgaacaagcctctcccgagccagcagaggaggaatcagtgatcgttcacatggcgtacaacaaggcgtgcggcgccgtcggctagcgctgtgaacgtcgcagcgttgattccaaccctctgcccacgacttcgccgctaactggaagcggtattaaacccgaaggttccacgggtggccacttggtgaaggcggcatcttaaagttctgacctcaattactatggtgccgtcatctaaccaaccattaagaagtagaattgtaaccggttatcggaatcagtgtcgacatactcaatgcccttaccctccgtaactctatgttttccgggttgttcatatcggacatgctatcgcttagacgactcgtctatatagggcgttagtatatcatgggcgtactatcccggtcaatcgtctacgtcgcaaatcacctccctttgggcaggagagcacttctacactgcctggcgctggcaccgcacaatcaataatgagattgtccggtgagacttacaatcccatcagcgagtctgcaatcccgctgtcacgataacatagtactcgccttctggcggttcctcagctatcctaacgtctatctaaactcaatgtggcgttatttggttcaagcaggcgggtcgtcaacgcgaaacgtaagtcccacacggccgtaacgcaatgtggcaattccagagttaccgatcgaggccaatggcccggccaagccgcaaccgcatcctccgccggtaaaatgactaaatgagtggaaccgtcgcgcttacgctctcggtgcccggaaaactctacagcatattgtctcattggtcctttggcgtatacagacacttagactaactctgactgtcgagtaaattccaatgaacgccagcactcagtacgcacggtgcgtccagggatggttacaaagctagagatgtatgtgctctcatcgaataacccgtagtatttggacctgaataccctggaatccgagggtgagaccattttacgttatcactgtgacatgctttttccggtcaatacacgggaaactacggacgctatggtcgggataaacgccgcatcatgtcaagctgaggccgctaaacaaagcatcaaggtagatttatttacaagcaaggccttgcgggcccagtccatgcgctgctgagattacagggattccgtgccagcccttcaacgtctcaagacaactaacaggccttgaattcggccacactcaccggtcccacaatgtgccgggttcgcatcaccgctgcttgggatagtatgcacacaaaagtagcttccacgagcggttgcccaattagatggcgacccgcgtacaggctctaggaggctggaaagtccctctcaccgtcaaatatctgagacgttatacccgacccatcatacgcgaataaagtactagtctcgcctgtcgccctcaggtttaccaccaataggacacggaaggcgctctgacaccagccaactagacagacatcggcgaggtgtacttgacgcactttagaagctgctcccttgtgggaaccattgggcaaccgaacatagccgcaatccagtcgcatcatcggtgggtcactgaccgaggattttggcggctcacgccttctgggctagcttcttctggcttgttagttattgcgtttacgttactctatatcccactaactatctatatacctgtgctttcactacaatggctgcacagttatcttattttagcaaagctttgggttgcctggagtttcccaaagcgggacttgtaagcccgtcctatatcaggaggcggacgagagacgcagcatgttatcttctaatctgcgaacggtacccggtctttcgtcggctaactagatctgtgtcctaggtattcaaatcgggcgaagtcggtatcgaagaaaagccctttaacgtaaactgtattcgtcggccgccgttagctcaatgatataactctcatcgcgtgagtcatggcgccacattttataaaacacgctactatccaatcgaggagatcgctgcaccaataacagtctcaatccaacgaactagtatttcatcaccgatccgcgaatcgtgagaccgacccatcgtcttatcgtcctaagcagcacgggccacgacgtgcgaggcggcgtaacttgcgagttctacctatatacttgacaatctcagctactccaaacgctgagagcagcactgtgcaagactggaaaggtggtgaagcaatacaccatgaatcccggatcgctgacgcgccctgtagcggcgcattaagcgcggcgggtgtggtggttacgcgcagcgtgaccgctacacttgccagcgccctagcgcccgctcctttcgctttcttcccttcctttctcgccacgttcgccggctttccccgtcaagctctaaatcgggggctccctttagggttccgatttagtgctttacggcacctcgaccccaaaaaacttgatttgggtgatggttcacgtagtgggccatcgccctgatagacggtttttcgccctttgacgttggagtccacgttcttt |
| 5040-nt ssDNA sequence for length test | aatagtggactcttgttccaaactggaacaacactcaaccctatctcgggctattcttttgatttataagggattttgccgatttcggaacgggtacctacgaagagttccagcagggattccaagaaatggccaatgaagattgtttcccgcacctgggctctttcgcgccaatcgacgttaaacacttgaatttaagatgagcatcgcagaagtggatgatatgcgcgcctacggtccaatcggctgggtgagcgtccctataatctcacggataccgacgaaagtagggcaataatcgcgctgcatgagaacggatagcccggatgcaaaggtggaacgtaattgttaagagaagcaaatacgttaagctatcatttcccgtaaatcttttacatacgtaaggtggaagtctaaactagcctagctgcacaagcatcggactgcttgccctctatctttgtggaagttcaaggtaatggaagcggtagaacgtatgatcgtcacgcagacctaaagaatcacgccgcgcttacgtccaaccttgggacctacatctgtacagacccaggtccaggccgaggttatggatgtagatttgacttggctgatctacagtctcgagttacaatggcgtcactgctacctccatatgaaggataagaggcagccacaactcagccaccatcctaccggaaacgctttaagggcgaacgtgaagggaagtgcctacacgaccctctatgtgggaagtagtctacgatacacgatgattggaagtgtggcggcataggatgcatctacggaccgacactagttatagaagggtgcagtccttgacgcatacgaatagcgttagctcgcgatttcccgctgggttactcatggagagcggctcagacgcctagcccatactgaccgtgtaccaacgatccaccaattattttcagaacagcccttttatggcgaagggaaaacagagccttagtatgttacagtccgctctattgggagatctcgggcaggcggaagatattaattagaaccgcagaccgcatgatgtgccgtcctcaaatggcctggatatcccgtcacacgaattatctcagttgaagtgatcctttagggcccgagagcaccacaagctcccaaccgctgcgaagagtgctaacgagttgtcctatgttaagttattcgtatttaacccacgcccgcaccacctccgggaataatgtccaagatcatatcaaggaaacccagggcacgataagcggcacacagacaaccactttggctagttaagataacggatactctgacgtacagccggccattcgttctacttttgtgtagtcagatggtccgtaacaatggcccggctctgacaacaagtataagactgcatgacgggtgggtccacgttagatgttcgtgaggtagcccatacatcattctttacgaacaagcctctcccgagccagcagaggaggaatcagtgatcgttcacatggcgtacaacaaggcgtgcggcgccgtcggctagcgctgtgaacgtcgcagcgttgattccaaccctctgcccacgacttcgccgctaactggaagcggtattaaacccgaaggttccacgggtggccacttggtgaaggcggcatcttaaagttctgacctcaattactatggtgccgtcatctaaccaaccattaagaagtagaattgtaaccggttatcggaatcagtgtcgacatactcaatgcccttaccctccgtaactctatgttttccgggttgttcatatcggacatgctatcgcttagacgactcgtctatatagggcgttagtatatcatgggcgtactatcccggtcaatcgtctacgtcgcaaatcacctccctttgggcaggagagcacttctacactgcctggcgctggcaccgcacaatcaataatgagattgtccggtgagacttacaatcccatcagcgagtctgcaatcccgctgtcacgataacatagtactcgccttctggcggttcctcagctatcctaacgtctatctaaactcaatgtggcgttatttggttcaagcaggcgggtcgtcaacgcgaaacgtaagtcccacacggccgtaacgcaatgtggcaattccagagttaccgatcgaggccaatggcccggccaagccgcaaccgcatcctccgccggtaaaatgactaaatgagtggaaccgtcgcgcttacgctctcggtgcccggaaaactctacagcatattgtctcattggtcctttggcgtatacagacacttagactaactctgactgtcgagtaaattccaatgaacgccagcactcagtacgcacggtgcgtccagggatggttacaaagctagagatgtatgtgctctcatcgaataacccgtagtatttggacctgaataccctggaatccgagggtgagaccattttacgttatcactgtgacatgctttttccggtcaatacacgggaaactacggacgctatggtcgggataaacgccgcatcatgtcaagctgaggccgctaaacaaagcatcaaggtagatttatttacaagcaaggccttgcgggcccagtccatgcgctgctgagattacagggattccgtgccagcccttcaacgtctcaagacaactaacaggccttgaattcggccacactcaccggtcccacaatgtgccgggttcgcatcaccgctgcttgggatagtatgcacacaaaagtagcttccacgagcggttgcccaattagatggcgacccgcgtacaggctctaggaggctggaaagtccctctcaccgtcaaatatctgagacgttatacccgacccatcatacgcgaataaagtactagtctcgcctgtcgccctcaggtttaccaccaataggacacggaaggcgctctgacaccagccaactagacagacatcggcgaggtgtacttgacgcactttagaagctgctcccttgtgggaaccattgggcaaccgaacatagccgcaatccagtcgcatcatcggtgggtcactgaccgaggattttggcggctcacgccttctgggctagcttcttctggcttgttagttattgcgtttacgttactctatatcccactaactatctatatacctgtgctttcactacaatggctgcacagttatcttattttagcaaagctttgggttgcctggagtttcccaaagcgggacttgtaagcccgtcctatatcaggaggcggacgagagacgcagcatgttatcttctaatctgcgaacggtacccggtctttcgtcggctaactagatctgtgtcctaggtattcaaatcgggcgaagtcggtatcgaagaaaagccctttaacgtaaactgtattcgtcggccgccgttagctcaatgatataactctcatcgcgtgagtcatggcgccacattttataaaacacgctactatccaatcgaggagatcgctgcaccaataacagtctcaatccaacgaactagtatttcatcaccgatccgcgaatcgtgagaccgacccatcgtcttatcgtcctaagcagcacgggccacgacgtgcgaggcggcgtaacttgcgagttctacctatatacttgacaatctcagctactccaaactgcccatggttggttggccatatgtacaccgagtcctagtacatcctcactggacacgcgttcgcttgttggagagatgaaatccaagatattccttgtagggagctaatcttgccaacactcaaattcctgatgcctcccaaaatacccgggctcaggtcaaaaaagccatgaagcttcaagcccatgcttttcttgagtgattatcgctggccgggcgtataagttaatccagctaactggcgtgtcaacgaaagggtgggacacaatggttttccggctgtctcccagcaagtgtcagaggcatttgcctttctctcaaccaaagcgctacactacacaggtcatcccgtgaacattaggtagagttctccagtcagtcctcgacacgagtccagccactcgaacttagtttaaggtcggcgagcaagaccaggtaacgagcaaccaatacatctgtcctttgacccggcatgtcctgctgtacaggtccgcattagatcagaagtgcggttccatgacgagccacgttccctacaacgaagcgtaaactagtacccttctacacaggcaccgcccggagtaggaaggattatgcttttgcctttaggaatttctagattctggtccgtgctgcggcctgcaacgtggacttacttataactgcggttaggacgattcatctgaaggaatacgctctttttcgactgcagctcgcgtgacgcttggctgaaaaattgaaactggagcttcctctacggatcaacgtttaactacccactgcctattcctatgtactgatcgtcgagtcttgccaggattccgtcgcgggactcgtatcaacgttcagttgagttgtgtcatgctaaccgcacattgtgagtcaccaagtgtcccatttggtcaactgatctcgcaaaaggtaagggccgtagcaaagtcgccagcttcgtcaattgatggcctatttttaactcgccgcttacggtcggaatctgaacggagacggctgagagcagcactgtgcaagactggaaaggtggtgaagcaatacaccatgaatcccggatcgctgacgcgccctgtagcggcgcattaagcgcggcgggtgtggtggttacgcgcagcgtgaccgctacacttgccagcgccctagcgcccgctcctttcgctttcttcccttcctttctcgccacgttcgccggctttccccgtcaagctctaaatcgggggctccctttagggttccgatttagtgctttacggcacctcgaccccaaaaaacttgatttgggtgatggttcacgtagtgggccatcgccctgatagacggtttttcgccctttgacgttggagtccacgttcttt |
| 6048-nt ssDNA sequence for length test | aatagtggactcttgttccaaactggaacaacactcaaccctatctcgggctattcttttgatttataagggattttgccgatttcggaacgggtacctacgaagagttccagcagggattccaagaaatggccaatgaagattgtttcccgcacctgggctctttcgcgccaatcgacgttaaacacttgaatttaagatgagcatcgcagaagtggatgatatgcgcgcctacggtccaatcggctgggtgagcgtccctataatctcacggataccgacgaaagtagggcaataatcgcgctgcatgagaacggatagcccggatgcaaaggtggaacgtaattgttaagagaagcaaatacgttaagctatcatttcccgtaaatcttttacatacgtaaggtggaagtctaaactagcctagctgcacaagcatcggactgcttgccctctatctttgtggaagttcaaggtaatggaagcggtagaacgtatgatcgtcacgcagacctaaagaatcacgccgcgcttacgtccaaccttgggacctacatctgtacagacccaggtccaggccgaggttatggatgtagatttgacttggctgatctacagtctcgagttacaatggcgtcactgctacctccatatgaaggataagaggcagccacaactcagccaccatcctaccggaaacgctttaagggcgaacgtgaagggaagtgcctacacgaccctctatgtgggaagtagtctacgatacacgatgattggaagtgtggcggcataggatgcatctacggaccgacactagttatagaagggtgcagtccttgacgcatacgaatagcgttagctcgcgatttcccgctgggttactcatggagagcggctcagacgcctagcccatactgaccgtgtaccaacgatccaccaattattttcagaacagcccttttatggcgaagggaaaacagagccttagtatgttacagtccgctctattgggagatctcgggcaggcggaagatattaattagaaccgcagaccgcatgatgtgccgtcctcaaatggcctggatatcccgtcacacgaattatctcagttgaagtgatcctttagggcccgagagcaccacaagctcccaaccgctgcgaagagtgctaacgagttgtcctatgttaagttattcgtatttaacccacgcccgcaccacctccgggaataatgtccaagatcatatcaaggaaacccagggcacgataagcggcacacagacaaccactttggctagttaagataacggatactctgacgtacagccggccattcgttctacttttgtgtagtcagatggtccgtaacaatggcccggctctgacaacaagtataagactgcatgacgggtgggtccacgttagatgttcgtgaggtagcccatacatcattctttacgaacaagcctctcccgagccagcagaggaggaatcagtgatcgttcacatggcgtacaacaaggcgtgcggcgccgtcggctagcgctgtgaacgtcgcagcgttgattccaaccctctgcccacgacttcgccgctaactggaagcggtattaaacccgaaggttccacgggtggccacttggtgaaggcggcatcttaaagttctgacctcaattactatggtgccgtcatctaaccaaccattaagaagtagaattgtaaccggttatcggaatcagtgtcgacatactcaatgcccttaccctccgtaactctatgttttccgggttgttcatatcggacatgctatcgcttagacgactcgtctatatagggcgttagtatatcatgggcgtactatcccggtcaatcgtctacgtcgcaaatcacctccctttgggcaggagagcacttctacactgcctggcgctggcaccgcacaatcaataatgagattgtccggtgagacttacaatcccatcagcgagtctgcaatcccgctgtcacgataacatagtactcgccttctggcggttcctcagctatcctaacgtctatctaaactcaatgtggcgttatttggttcaagcaggcgggtcgtcaacgcgaaacgtaagtcccacacggccgtaacgcaatgtggcaattccagagttaccgatcgaggccaatggcccggccaagccgcaaccgcatcctccgccggtaaaatgactaaatgagtggaaccgtcgcgcttacgctctcggtgcccggaaaactctacagcatattgtctcattggtcctttggcgtatacagacacttagactaactctgactgtcgagtaaattccaatgaacgccagcactcagtacgcacggtgcgtccagggatggttacaaagctagagatgtatgtgctctcatcgaataacccgtagtatttggacctgaataccctggaatccgagggtgagaccattttacgttatcactgtgacatgctttttccggtcaatacacgggaaactacggacgctatggtcgggataaacgccgcatcatgtcaagctgaggccgctaaacaaagcatcaaggtagatttatttacaagcaaggccttgcgggcccagtccatgcgctgctgagattacagggattccgtgccagcccttcaacgtctcaagacaactaacaggccttgaattcggccacactcaccggtcccacaatgtgccgggttcgcatcaccgctgcttgggatagtatgcacacaaaagtagcttccacgagcggttgcccaattagatggcgacccgcgtacaggctctaggaggctggaaagtccctctcaccgtcaaatatctgagacgttatacccgacccatcatacgcgaataaagtactagtctcgcctgtcgccctcaggtttaccaccaataggacacggaaggcgctctgacaccagccaactagacagacatcggcgaggtgtacttgacgcactttagaagctgctcccttgtgggaaccattgggcaaccgaacatagccgcaatccagtcgcatcatcggtgggtcactgaccgaggattttggcggctcacgccttctgggctagcttcttctggcttgttagttattgcgtttacgttactctatatcccactaactatctatatacctgtgctttcactacaatggctgcacagttatcttattttagcaaagctttgggttgcctggagtttcccaaagcgggacttgtaagcccgtcctatatcaggaggcggacgagagacgcagcatgttatcttctaatctgcgaacggtacccggtctttcgtcggctaactagatctgtgtcctaggtattcaaatcgggcgaagtcggtatcgaagaaaagccctttaacgtaaactgtattcgtcggccgccgttagctcaatgatataactctcatcgcgtgagtcatggcgccacattttataaaacacgctactatccaatcgaggagatcgctgcaccaataacagtctcaatccaacgaactagtatttcatcaccgatccgcgaatcgtgagaccgacccatcgtcttatcgtcctaagcagcacgggccacgacgtgcgaggcggcgtaacttgcgagttctacctatatacttgacaatctcagctactccaaactgcccatggttggttggccatatgtacaccgagtcctagtacatcctcactggacacgcgttcgcttgttggagagatgaaatccaagatattccttgtagggagctaatcttgccaacactcaaattcctgatgcctcccaaaatacccgggctcaggtcaaaaaagccatgaagcttcaagcccatgcttttcttgagtgattatcgctggccgggcgtataagttaatccagctaactggcgtgtcaacgaaagggtgggacacaatggttttccggctgtctcccagcaagtgtcagaggcatttgcctttctctcaaccaaagcgctacactacacaggtcatcccgtgaacattaggtagagttctccagtcagtcctcgacacgagtccagccactcgaacttagtttaaggtcggcgagcaagaccaggtaacgagcaaccaatacatctgtcctttgacccggcatgtcctgctgtacaggtccgcattagatcagaagtgcggttccatgacgagccacgttccctacaacgaagcgtaaactagtacccttctacacaggcaccgcccggagtaggaaggattatgcttttgcctttaggaatttctagattctggtccgtgctgcggcctgcaacgtggacttacttataactgcggttaggacgattcatctgaaggaatacgctctttttcgactgcagctcgcgtgacgcttggctgaaaaattgaaactggagcttcctctacggatcaacgtttaactacccactgcctattcctatgtactgatcgtcgagtcttgccaggattccgtcgcgggactcgtatcaacgttcagttgagttgtgtcatgctaaccgcacattgtgagtcaccaagtgtcccatttggtcaactgatctcgcaaaaggtaagggccgtagcaaagtcgccagcttcgtcaattgatggcctatttttaactcgccgcttacggtcggaatctgaacggagacgtctttggcagaatggcgttacgcaccaatctataaaaagtttttgttggaaaggaggataatttctactggaccggtgttgcgacggaggagatcgaattgctaatcaaccggtatgcacacttccattgctgtgcagttgccctaatcagctatccatccaacataaaactgtctgagtgcttaaacggtcacaccaaatgattgtggtgccgtatctatagaatatccttagagcgtctgcttcctcgtcacacgagaccggttagtcccaagcacaataacgaatccagcttctgtttgccttagctccggtgatgcatgtttctgcttccggcggtgcggatgccacagctgccactgcaggtggaggagaagctgccaagtccaaaccaactacatttactccaccagattccacccatatcgacttacttccaatagctggttttcgcgttgacccacattacaatgtatctacgactcaagttattttaatgtatagcgttctgtattcgacccttccataatgccctctatgtgaaactaacaacaatttgaccctcaaactttaagtataccagcttatggcaacagtctgcgagccatggaagggatataacctgacgcagattattgcactgtccaagatccttcatgccacatcttcaggagggcgggtgattagcaccgtaaacagcgggttgatatcatcaagcgaactgcagagaaatccgcgggaacactgggcttagcgcccatctcacccttaaaaattaaacgcatctcccggtttcaggcattgctaccctgcgccggctagcgccttcccaatcctgtggcttaagtctactgcgaaacaggttttataacagttccaccgcaatcaggtggccatttgtcctcactctaatcccatccacccgttgatagtcaaagattcctctaataggcccatgaacgtgcaaagttcccaatcgaacccacttggcacatacagtatccggcagctgagagcagcactgtgcaagactggaaaggtggtgaagcaatacaccatgaatcccggatcgctgacgcgccctgtagcggcgcattaagcgcggcgggtgtggtggttacgcgcagcgtgaccgctacacttgccagcgccctagcgcccgctcctttcgctttcttcccttcctttctcgccacgttcgccggctttccccgtcaagctctaaatcgggggctccctttagggttccgatttagtgctttacggcacctcgaccccaaaaaacttgatttgggtgatggttcacgtagtgggccatcgccctgatagacggtttttcgccctttgacgttggagtccacgttcttt |
| 7056-nt ssDNA sequence for length test | aatagtggactcttgttccaaactggaacaacactcaaccctatctcgggctattcttttgatttataagggattttgccgatttcggaacgggtacctacgaagagttccagcagggattccaagaaatggccaatgaagattgtttcccgcacctgggctctttcgcgccaatcgacgttaaacacttgaatttaagatgagcatcgcagaagtggatgatatgcgcgcctacggtccaatcggctgggtgagcgtccctataatctcacggataccgacgaaagtagggcaataatcgcgctgcatgagaacggatagcccggatgcaaaggtggaacgtaattgttaagagaagcaaatacgttaagctatcatttcccgtaaatcttttacatacgtaaggtggaagtctaaactagcctagctgcacaagcatcggactgcttgccctctatctttgtggaagttcaaggtaatggaagcggtagaacgtatgatcgtcacgcagacctaaagaatcacgccgcgcttacgtccaaccttgggacctacatctgtacagacccaggtccaggccgaggttatggatgtagatttgacttggctgatctacagtctcgagttacaatggcgtcactgctacctccatatgaaggataagaggcagccacaactcagccaccatcctaccggaaacgctttaagggcgaacgtgaagggaagtgcctacacgaccctctatgtgggaagtagtctacgatacacgatgattggaagtgtggcggcataggatgcatctacggaccgacactagttatagaagggtgcagtccttgacgcatacgaatagcgttagctcgcgatttcccgctgggttactcatggagagcggctcagacgcctagcccatactgaccgtgtaccaacgatccaccaattattttcagaacagcccttttatggcgaagggaaaacagagccttagtatgttacagtccgctctattgggagatctcgggcaggcggaagatattaattagaaccgcagaccgcatgatgtgccgtcctcaaatggcctggatatcccgtcacacgaattatctcagttgaagtgatcctttagggcccgagagcaccacaagctcccaaccgctgcgaagagtgctaacgagttgtcctatgttaagttattcgtatttaacccacgcccgcaccacctccgggaataatgtccaagatcatatcaaggaaacccagggcacgataagcggcacacagacaaccactttggctagttaagataacggatactctgacgtacagccggccattcgttctacttttgtgtagtcagatggtccgtaacaatggcccggctctgacaacaagtataagactgcatgacgggtgggtccacgttagatgttcgtgaggtagcccatacatcattctttacgaacaagcctctcccgagccagcagaggaggaatcagtgatcgttcacatggcgtacaacaaggcgtgcggcgccgtcggctagcgctgtgaacgtcgcagcgttgattccaaccctctgcccacgacttcgccgctaactggaagcggtattaaacccgaaggttccacgggtggccacttggtgaaggcggcatcttaaagttctgacctcaattactatggtgccgtcatctaaccaaccattaagaagtagaattgtaaccggttatcggaatcagtgtcgacatactcaatgcccttaccctccgtaactctatgttttccgggttgttcatatcggacatgctatcgcttagacgactcgtctatatagggcgttagtatatcatgggcgtactatcccggtcaatcgtctacgtcgcaaatcacctccctttgggcaggagagcacttctacactgcctggcgctggcaccgcacaatcaataatgagattgtccggtgagacttacaatcccatcagcgagtctgcaatcccgctgtcacgataacatagtactcgccttctggcggttcctcagctatcctaacgtctatctaaactcaatgtggcgttatttggttcaagcaggcgggtcgtcaacgcgaaacgtaagtcccacacggccgtaacgcaatgtggcaattccagagttaccgatcgaggccaatggcccggccaagccgcaaccgcatcctccgccggtaaaatgactaaatgagtggaaccgtcgcgcttacgctctcggtgcccggaaaactctacagcatattgtctcattggtcctttggcgtatacagacacttagactaactctgactgtcgagtaaattccaatgaacgccagcactcagtacgcacggtgcgtccagggatggttacaaagctagagatgtatgtgctctcatcgaataacccgtagtatttggacctgaataccctggaatccgagggtgagaccattttacgttatcactgtgacatgctttttccggtcaatacacgggaaactacggacgctatggtcgggataaacgccgcatcatgtcaagctgaggccgctaaacaaagcatcaaggtagatttatttacaagcaaggccttgcgggcccagtccatgcgctgctgagattacagggattccgtgccagcccttcaacgtctcaagacaactaacaggccttgaattcggccacactcaccggtcccacaatgtgccgggttcgcatcaccgctgcttgggatagtatgcacacaaaagtagcttccacgagcggttgcccaattagatggcgacccgcgtacaggctctaggaggctggaaagtccctctcaccgtcaaatatctgagacgttatacccgacccatcatacgcgaataaagtactagtctcgcctgtcgccctcaggtttaccaccaataggacacggaaggcgctctgacaccagccaactagacagacatcggcgaggtgtacttgacgcactttagaagctgctcccttgtgggaaccattgggcaaccgaacatagccgcaatccagtcgcatcatcggtgggtcactgaccgaggattttggcggctcacgccttctgggctagcttcttctggcttgttagttattgcgtttacgttactctatatcccactaactatctatatacctgtgctttcactacaatggctgcacagttatcttattttagcaaagctttgggttgcctggagtttcccaaagcgggacttgtaagcccgtcctatatcaggaggcggacgagagacgcagcatgttatcttctaatctgcgaacggtacccggtctttcgtcggctaactagatctgtgtcctaggtattcaaatcgggcgaagtcggtatcgaagaaaagccctttaacgtaaactgtattcgtcggccgccgttagctcaatgatataactctcatcgcgtgagtcatggcgccacattttataaaacacgctactatccaatcgaggagatcgctgcaccaataacagtctcaatccaacgaactagtatttcatcaccgatccgcgaatcgtgagaccgacccatcgtcttatcgtcctaagcagcacgggccacgacgtgcgaggcggcgtaacttgcgagttctacctatatacttgacaatctcagctactccaaactgcccatggttggttggccatatgtacaccgagtcctagtacatcctcactggacacgcgttcgcttgttggagagatgaaatccaagatattccttgtagggagctaatcttgccaacactcaaattcctgatgcctcccaaaatacccgggctcaggtcaaaaaagccatgaagcttcaagcccatgcttttcttgagtgattatcgctggccgggcgtataagttaatccagctaactggcgtgtcaacgaaagggtgggacacaatggttttccggctgtctcccagcaagtgtcagaggcatttgcctttctctcaaccaaagcgctacactacacaggtcatcccgtgaacattaggtagagttctccagtcagtcctcgacacgagtccagccactcgaacttagtttaaggtcggcgagcaagaccaggtaacgagcaaccaatacatctgtcctttgacccggcatgtcctgctgtacaggtccgcattagatcagaagtgcggttccatgacgagccacgttccctacaacgaagcgtaaactagtacccttctacacaggcaccgcccggagtaggaaggattatgcttttgcctttaggaatttctagattctggtccgtgctgcggcctgcaacgtggacttacttataactgcggttaggacgattcatctgaaggaatacgctctttttcgactgcagctcgcgtgacgcttggctgaaaaattgaaactggagcttcctctacggatcaacgtttaactacccactgcctattcctatgtactgatcgtcgagtcttgccaggattccgtcgcgggactcgtatcaacgttcagttgagttgtgtcatgctaaccgcacattgtgagtcaccaagtgtcccatttggtcaactgatctcgcaaaaggtaagggccgtagcaaagtcgccagcttcgtcaattgatggcctatttttaactcgccgcttacggtcggaatctgaacggagacgtctttggcagaatggcgttacgcaccaatctataaaaagtttttgttggaaaggaggataatttctactggaccggtgttgcgacggaggagatcgaattgctaatcaaccggtatgcacacttccattgctgtgcagttgccctaatcagctatccatccaacataaaactgtctgagtgcttaaacggtcacaccaaatgattgtggtgccgtatctatagaatatccttagagcgtctgcttcctcgtcacacgagaccggttagtcccaagcacaataacgaatccagcttctgtttgccttagctccggtgatgcatgtttctgcttccggcggtgcggatgccacagctgccactgcaggtggaggagaagctgccaagtccaaaccaactacatttactccaccagattccacccatatcgacttacttccaatagctggttttcgcgttgacccacattacaatgtatctacgactcaagttattttaatgtatagcgttctgtattcgacccttccataatgccctctatgtgaaactaacaacaatttgaccctcaaactttaagtataccagcttatggcaacagtctgcgagccatggaagggatataacctgacgcagattattgcactgtccaagatccttcatgccacatcttcaggagggcgggtgattagcaccgtaaacagcgggttgatatcatcaagcgaactgcagagaaatccgcgggaacactgggcttagcgcccatctcacccttaaaaattaaacgcatctcccggtttcaggcattgctaccctgcgccggctagcgccttcccaatcctgtggcttaagtctactgcgaaacaggttttataacagttccaccgcaatcaggtggccatttgtcctcactctaatcccatccacccgttgatagtcaaagattcctctaataggcccatgaacgtgcaaagttcccaatcgaacccacttggcacatacagtatccggcaaatgttatatcaacaagtcgcctgaacgtgccgcaacaacggatcaactgtagcttcgtgctgcctcagatgcatggctcgtgcccttctgttcgtgctgcatcggttgtatctcaatgactggactcagcatcgtagctaagtaggcagggattttagtggtgtctaaagaataccggcggtaggcccaaaaaattacctcgttcacccactaaagagatatcccgtacatctactatctactcaggaagatcaccactctagcgtgggagccgctataatggatgcaggcagcccgggttagcgtgatgaaggacgttttaagttactactactggagttgcgggcgcaagacgatggctaagtaagagcccagagtttaggccttgtctaaaccgtaatgaaactgacatcggtagtcaatgtgtcgacgagttttgatttcagtatatacgtactgttaaccgacgtctggatgtcagaaatttcgtgcatgtggcaggctcggttcgaggaatctcgttccggaagttagggtatcggcgagggaactagtataaggactcgactgatgcatggctcagctaacagcgggcactctatgtcctaaggatagtaagaggagcaggacaaccatccgggtgtaacgggttgatgcaagcgacatactaacaatgcctaggatagctgtgccatcaggcggaaaagaatccaatatgatggtgctcaggactcattattacaatatagtacatttaccagagaggtcccgcgggtcgccgaacacctacgcgaccctataagtttcttacactatcgataatggagaaagcttatttgagggactacgatcttttacacccatggactttcagccgagatatcaaaatcgtagttatgttgtagcctgtagatttgtattcacgggctgtactttagccgaggacagacccatatctgcttataataggtcatcatccctacattgtgtgagccagtctccaccgcgctgagagcagcactgtgcaagactggaaaggtggtgaagcaatacaccatgaatcccggatcgctgacgcgccctgtagcggcgcattaagcgcggcgggtgtggtggttacgcgcagcgtgaccgctacacttgccagcgccctagcgcccgctcctttcgctttcttcccttcctttctcgccacgttcgccggctttccccgtcaagctctaaatcgggggctccctttagggttccgatttagtgctttacggcacctcgaccccaaaaaacttgatttgggtgatggttcacgtagtgggccatcgccctgatagacggtttttcgccctttgacgttggagtccacgttcttt |
| 8064-nt ssDNA sequence for length test | aatagtggactcttgttccaaactggaacaacactcaaccctatctcgggctattcttttgatttataagggattttgccgatttcggaacgggtacctacgaagagttccagcagggattccaagaaatggccaatgaagattgtttcccgcacctgggctctttcgcgccaatcgacgttaaacacttgaatttaagatgagcatcgcagaagtggatgatatgcgcgcctacggtccaatcggctgggtgagcgtccctataatctcacggataccgacgaaagtagggcaataatcgcgctgcatgagaacggatagcccggatgcaaaggtggaacgtaattgttaagagaagcaaatacgttaagctatcatttcccgtaaatcttttacatacgtaaggtggaagtctaaactagcctagctgcacaagcatcggactgcttgccctctatctttgtggaagttcaaggtaatggaagcggtagaacgtatgatcgtcacgcagacctaaagaatcacgccgcgcttacgtccaaccttgggacctacatctgtacagacccaggtccaggccgaggttatggatgtagatttgacttggctgatctacagtctcgagttacaatggcgtcactgctacctccatatgaaggataagaggcagccacaactcagccaccatcctaccggaaacgctttaagggcgaacgtgaagggaagtgcctacacgaccctctatgtgggaagtagtctacgatacacgatgattggaagtgtggcggcataggatgcatctacggaccgacactagttatagaagggtgcagtccttgacgcatacgaatagcgttagctcgcgatttcccgctgggttactcatggagagcggctcagacgcctagcccatactgaccgtgtaccaacgatccaccaattattttcagaacagcccttttatggcgaagggaaaacagagccttagtatgttacagtccgctctattgggagatctcgggcaggcggaagatattaattagaaccgcagaccgcatgatgtgccgtcctcaaatggcctggatatcccgtcacacgaattatctcagttgaagtgatcctttagggcccgagagcaccacaagctcccaaccgctgcgaagagtgctaacgagttgtcctatgttaagttattcgtatttaacccacgcccgcaccacctccgggaataatgtccaagatcatatcaaggaaacccagggcacgataagcggcacacagacaaccactttggctagttaagataacggatactctgacgtacagccggccattcgttctacttttgtgtagtcagatggtccgtaacaatggcccggctctgacaacaagtataagactgcatgacgggtgggtccacgttagatgttcgtgaggtagcccatacatcattctttacgaacaagcctctcccgagccagcagaggaggaatcagtgatcgttcacatggcgtacaacaaggcgtgcggcgccgtcggctagcgctgtgaacgtcgcagcgttgattccaaccctctgcccacgacttcgccgctaactggaagcggtattaaacccgaaggttccacgggtggccacttggtgaaggcggcatcttaaagttctgacctcaattactatggtgccgtcatctaaccaaccattaagaagtagaattgtaaccggttatcggaatcagtgtcgacatactcaatgcccttaccctccgtaactctatgttttccgggttgttcatatcggacatgctatcgcttagacgactcgtctatatagggcgttagtatatcatgggcgtactatcccggtcaatcgtctacgtcgcaaatcacctccctttgggcaggagagcacttctacactgcctggcgctggcaccgcacaatcaataatgagattgtccggtgagacttacaatcccatcagcgagtctgcaatcccgctgtcacgataacatagtactcgccttctggcggttcctcagctatcctaacgtctatctaaactcaatgtggcgttatttggttcaagcaggcgggtcgtcaacgcgaaacgtaagtcccacacggccgtaacgcaatgtggcaattccagagttaccgatcgaggccaatggcccggccaagccgcaaccgcatcctccgccggtaaaatgactaaatgagtggaaccgtcgcgcttacgctctcggtgcccggaaaactctacagcatattgtctcattggtcctttggcgtatacagacacttagactaactctgactgtcgagtaaattccaatgaacgccagcactcagtacgcacggtgcgtccagggatggttacaaagctagagatgtatgtgctctcatcgaataacccgtagtatttggacctgaataccctggaatccgagggtgagaccattttacgttatcactgtgacatgctttttccggtcaatacacgggaaactacggacgctatggtcgggataaacgccgcatcatgtcaagctgaggccgctaaacaaagcatcaaggtagatttatttacaagcaaggccttgcgggcccagtccatgcgctgctgagattacagggattccgtgccagcccttcaacgtctcaagacaactaacaggccttgaattcggccacactcaccggtcccacaatgtgccgggttcgcatcaccgctgcttgggatagtatgcacacaaaagtagcttccacgagcggttgcccaattagatggcgacccgcgtacaggctctaggaggctggaaagtccctctcaccgtcaaatatctgagacgttatacccgacccatcatacgcgaataaagtactagtctcgcctgtcgccctcaggtttaccaccaataggacacggaaggcgctctgacaccagccaactagacagacatcggcgaggtgtacttgacgcactttagaagctgctcccttgtgggaaccattgggcaaccgaacatagccgcaatccagtcgcatcatcggtgggtcactgaccgaggattttggcggctcacgccttctgggctagcttcttctggcttgttagttattgcgtttacgttactctatatcccactaactatctatatacctgtgctttcactacaatggctgcacagttatcttattttagcaaagctttgggttgcctggagtttcccaaagcgggacttgtaagcccgtcctatatcaggaggcggacgagagacgcagcatgttatcttctaatctgcgaacggtacccggtctttcgtcggctaactagatctgtgtcctaggtattcaaatcgggcgaagtcggtatcgaagaaaagccctttaacgtaaactgtattcgtcggccgccgttagctcaatgatataactctcatcgcgtgagtcatggcgccacattttataaaacacgctactatccaatcgaggagatcgctgcaccaataacagtctcaatccaacgaactagtatttcatcaccgatccgcgaatcgtgagaccgacccatcgtcttatcgtcctaagcagcacgggccacgacgtgcgaggcggcgtaacttgcgagttctacctatatacttgacaatctcagctactccaaactgcccatggttggttggccatatgtacaccgagtcctagtacatcctcactggacacgcgttcgcttgttggagagatgaaatccaagatattccttgtagggagctaatcttgccaacactcaaattcctgatgcctcccaaaatacccgggctcaggtcaaaaaagccatgaagcttcaagcccatgcttttcttgagtgattatcgctggccgggcgtataagttaatccagctaactggcgtgtcaacgaaagggtgggacacaatggttttccggctgtctcccagcaagtgtcagaggcatttgcctttctctcaaccaaagcgctacactacacaggtcatcccgtgaacattaggtagagttctccagtcagtcctcgacacgagtccagccactcgaacttagtttaaggtcggcgagcaagaccaggtaacgagcaaccaatacatctgtcctttgacccggcatgtcctgctgtacaggtccgcattagatcagaagtgcggttccatgacgagccacgttccctacaacgaagcgtaaactagtacccttctacacaggcaccgcccggagtaggaaggattatgcttttgcctttaggaatttctagattctggtccgtgctgcggcctgcaacgtggacttacttataactgcggttaggacgattcatctgaaggaatacgctctttttcgactgcagctcgcgtgacgcttggctgaaaaattgaaactggagcttcctctacggatcaacgtttaactacccactgcctattcctatgtactgatcgtcgagtcttgccaggattccgtcgcgggactcgtatcaacgttcagttgagttgtgtcatgctaaccgcacattgtgagtcaccaagtgtcccatttggtcaactgatctcgcaaaaggtaagggccgtagcaaagtcgccagcttcgtcaattgatggcctatttttaactcgccgcttacggtcggaatctgaacggagacgtctttggcagaatggcgttacgcaccaatctataaaaagtttttgttggaaaggaggataatttctactggaccggtgttgcgacggaggagatcgaattgctaatcaaccggtatgcacacttccattgctgtgcagttgccctaatcagctatccatccaacataaaactgtctgagtgcttaaacggtcacaccaaatgattgtggtgccgtatctatagaatatccttagagcgtctgcttcctcgtcacacgagaccggttagtcccaagcacaataacgaatccagcttctgtttgccttagctccggtgatgcatgtttctgcttccggcggtgcggatgccacagctgccactgcaggtggaggagaagctgccaagtccaaaccaactacatttactccaccagattccacccatatcgacttacttccaatagctggttttcgcgttgacccacattacaatgtatctacgactcaagttattttaatgtatagcgttctgtattcgacccttccataatgccctctatgtgaaactaacaacaatttgaccctcaaactttaagtataccagcttatggcaacagtctgcgagccatggaagggatataacctgacgcagattattgcactgtccaagatccttcatgccacatcttcaggagggcgggtgattagcaccgtaaacagcgggttgatatcatcaagcgaactgcagagaaatccgcgggaacactgggcttagcgcccatctcacccttaaaaattaaacgcatctcccggtttcaggcattgctaccctgcgccggctagcgccttcccaatcctgtggcttaagtctactgcgaaacaggttttataacagttccaccgcaatcaggtggccatttgtcctcactctaatcccatccacccgttgatagtcaaagattcctctaataggcccatgaacgtgcaaagttcccaatcgaacccacttggcacatacagtatccggcaaatgttatatcaacaagtcgcctgaacgtgccgcaacaacggatcaactgtagcttcgtgctgcctcagatgcatggctcgtgcccttctgttcgtgctgcatcggttgtatctcaatgactggactcagcatcgtagctaagtaggcagggattttagtggtgtctaaagaataccggcggtaggcccaaaaaattacctcgttcacccactaaagagatatcccgtacatctactatctactcaggaagatcaccactctagcgtgggagccgctataatggatgcaggcagcccgggttagcgtgatgaaggacgttttaagttactactactggagttgcgggcgcaagacgatggctaagtaagagcccagagtttaggccttgtctaaaccgtaatgaaactgacatcggtagtcaatgtgtcgacgagttttgatttcagtatatacgtactgttaaccgacgtctggatgtcagaaatttcgtgcatgtggcaggctcggttcgaggaatctcgttccggaagttagggtatcggcgagggaactagtataaggactcgactgatgcatggctcagctaacagcgggcactctatgtcctaaggatagtaagaggagcaggacaaccatccgggtgtaacgggttgatgcaagcgacatactaacaatgcctaggatagctgtgccatcaggcggaaaagaatccaatatgatggtgctcaggactcattattacaatatagtacatttaccagagaggtcccgcgggtcgccgaacacctacgcgaccctataagtttcttacactatcgataatggagaaagcttatttgagggactacgatcttttacacccatggactttcagccgagatatcaaaatcgtagttatgttgtagcctgtagatttgtattcacgggctgtactttagccgaggacagacccatatctgcttataataggtcatcatccctacattgtgtgagccagtctccaccgctcgatgccaggcagtccaatgttatgtaaaacgaaggcgaaagggtgtctaacaccatctgatcgatacaaactcgcactggccgcccacaaacgtcatggaaaggtagaaaattcgatgggctgacgctattaccgtatcataggtcgactcacgtgggtacgtgccgtagctcccatgtttaccgcttatgcgggtccagtggaaagagctttactggacgaataacgctgctgctttttaatccatatatgatactgcctagaacaagtacgggaaaatttcgacggcgtgcaatgtgcaatatgtttccgctatttgatcactcttggccgagtgcaatctctcactcgcgcttttgggctaacgacatagactcaatatcttagagtgagacgtgcggtctttcagtggagaaagccctgtttagaccacaggttcctattattcggatgaacgacctttaagataggtcaaccattatgacagttgcctgagtaagaacacgagcggagtatctgattctgatgcttagacgctgtcgcatcccgtgaaagctcatccagaccgggtgagcgtagacctataactacgccaacctacccggccgggaaatcatgtaggcaactcaacccgctcgcatgtaagttgtccataatatgaatttacccatccgaattgtatcgtggagttgttcggctagtggcaggagttcttagactaatgacaccctcactgttgcggcggtaacaaccattaactattacaagttgcggtttggtaaggttagggtaactgtagttaaaagtatttctgcgattgctcgtttcgtcagatcacttcacagcgcagtctacggcactagggacaagatttgtgtactgtggacccgtagccgagaaatccacggcattcatgagacgttactcgggaactattcagtcagtgtatgtagtcggcaaccggtagtggttccggaacaagcttttgaaaatcagttaatgtggggctgagagcagcactgtgcaagactggaaaggtggtgaagcaatacaccatgaatcccggatcgctgacgcgccctgtagcggcgcattaagcgcggcgggtgtggtggttacgcgcagcgtgaccgctacacttgccagcgccctagcgcccgctcctttcgctttcttcccttcctttctcgccacgttcgccggctttccccgtcaagctctaaatcgggggctccctttagggttccgatttagtgctttacggcacctcgaccccaaaaaacttgatttgggtgatggttcacgtagtgggccatcgccctgatagacggtttttcgccctttgacgttggagtccacgttcttt |
| 9072-nt ssDNA sequence for length test | aatagtggactcttgttccaaactggaacaacactcaaccctatctcgggctattcttttgatttataagggattttgccgatttcggaacgggtacctacgaagagttccagcagggattccaagaaatggccaatgaagattgtttcccgcacctgggctctttcgcgccaatcgacgttaaacacttgaatttaagatgagcatcgcagaagtggatgatatgcgcgcctacggtccaatcggctgggtgagcgtccctataatctcacggataccgacgaaagtagggcaataatcgcgctgcatgagaacggatagcccggatgcaaaggtggaacgtaattgttaagagaagcaaatacgttaagctatcatttcccgtaaatcttttacatacgtaaggtggaagtctaaactagcctagctgcacaagcatcggactgcttgccctctatctttgtggaagttcaaggtaatggaagcggtagaacgtatgatcgtcacgcagacctaaagaatcacgccgcgcttacgtccaaccttgggacctacatctgtacagacccaggtccaggccgaggttatggatgtagatttgacttggctgatctacagtctcgagttacaatggcgtcactgctacctccatatgaaggataagaggcagccacaactcagccaccatcctaccggaaacgctttaagggcgaacgtgaagggaagtgcctacacgaccctctatgtgggaagtagtctacgatacacgatgattggaagtgtggcggcataggatgcatctacggaccgacactagttatagaagggtgcagtccttgacgcatacgaatagcgttagctcgcgatttcccgctgggttactcatggagagcggctcagacgcctagcccatactgaccgtgtaccaacgatccaccaattattttcagaacagcccttttatggcgaagggaaaacagagccttagtatgttacagtccgctctattgggagatctcgggcaggcggaagatattaattagaaccgcagaccgcatgatgtgccgtcctcaaatggcctggatatcccgtcacacgaattatctcagttgaagtgatcctttagggcccgagagcaccacaagctcccaaccgctgcgaagagtgctaacgagttgtcctatgttaagttattcgtatttaacccacgcccgcaccacctccgggaataatgtccaagatcatatcaaggaaacccagggcacgataagcggcacacagacaaccactttggctagttaagataacggatactctgacgtacagccggccattcgttctacttttgtgtagtcagatggtccgtaacaatggcccggctctgacaacaagtataagactgcatgacgggtgggtccacgttagatgttcgtgaggtagcccatacatcattctttacgaacaagcctctcccgagccagcagaggaggaatcagtgatcgttcacatggcgtacaacaaggcgtgcggcgccgtcggctagcgctgtgaacgtcgcagcgttgattccaaccctctgcccacgacttcgccgctaactggaagcggtattaaacccgaaggttccacgggtggccacttggtgaaggcggcatcttaaagttctgacctcaattactatggtgccgtcatctaaccaaccattaagaagtagaattgtaaccggttatcggaatcagtgtcgacatactcaatgcccttaccctccgtaactctatgttttccgggttgttcatatcggacatgctatcgcttagacgactcgtctatatagggcgttagtatatcatgggcgtactatcccggtcaatcgtctacgtcgcaaatcacctccctttgggcaggagagcacttctacactgcctggcgctggcaccgcacaatcaataatgagattgtccggtgagacttacaatcccatcagcgagtctgcaatcccgctgtcacgataacatagtactcgccttctggcggttcctcagctatcctaacgtctatctaaactcaatgtggcgttatttggttcaagcaggcgggtcgtcaacgcgaaacgtaagtcccacacggccgtaacgcaatgtggcaattccagagttaccgatcgaggccaatggcccggccaagccgcaaccgcatcctccgccggtaaaatgactaaatgagtggaaccgtcgcgcttacgctctcggtgcccggaaaactctacagcatattgtctcattggtcctttggcgtatacagacacttagactaactctgactgtcgagtaaattccaatgaacgccagcactcagtacgcacggtgcgtccagggatggttacaaagctagagatgtatgtgctctcatcgaataacccgtagtatttggacctgaataccctggaatccgagggtgagaccattttacgttatcactgtgacatgctttttccggtcaatacacgggaaactacggacgctatggtcgggataaacgccgcatcatgtcaagctgaggccgctaaacaaagcatcaaggtagatttatttacaagcaaggccttgcgggcccagtccatgcgctgctgagattacagggattccgtgccagcccttcaacgtctcaagacaactaacaggccttgaattcggccacactcaccggtcccacaatgtgccgggttcgcatcaccgctgcttgggatagtatgcacacaaaagtagcttccacgagcggttgcccaattagatggcgacccgcgtacaggctctaggaggctggaaagtccctctcaccgtcaaatatctgagacgttatacccgacccatcatacgcgaataaagtactagtctcgcctgtcgccctcaggtttaccaccaataggacacggaaggcgctctgacaccagccaactagacagacatcggcgaggtgtacttgacgcactttagaagctgctcccttgtgggaaccattgggcaaccgaacatagccgcaatccagtcgcatcatcggtgggtcactgaccgaggattttggcggctcacgccttctgggctagcttcttctggcttgttagttattgcgtttacgttactctatatcccactaactatctatatacctgtgctttcactacaatggctgcacagttatcttattttagcaaagctttgggttgcctggagtttcccaaagcgggacttgtaagcccgtcctatatcaggaggcggacgagagacgcagcatgttatcttctaatctgcgaacggtacccggtctttcgtcggctaactagatctgtgtcctaggtattcaaatcgggcgaagtcggtatcgaagaaaagccctttaacgtaaactgtattcgtcggccgccgttagctcaatgatataactctcatcgcgtgagtcatggcgccacattttataaaacacgctactatccaatcgaggagatcgctgcaccaataacagtctcaatccaacgaactagtatttcatcaccgatccgcgaatcgtgagaccgacccatcgtcttatcgtcctaagcagcacgggccacgacgtgcgaggcggcgtaacttgcgagttctacctatatacttgacaatctcagctactccaaactgcccatggttggttggccatatgtacaccgagtcctagtacatcctcactggacacgcgttcgcttgttggagagatgaaatccaagatattccttgtagggagctaatcttgccaacactcaaattcctgatgcctcccaaaatacccgggctcaggtcaaaaaagccatgaagcttcaagcccatgcttttcttgagtgattatcgctggccgggcgtataagttaatccagctaactggcgtgtcaacgaaagggtgggacacaatggttttccggctgtctcccagcaagtgtcagaggcatttgcctttctctcaaccaaagcgctacactacacaggtcatcccgtgaacattaggtagagttctccagtcagtcctcgacacgagtccagccactcgaacttagtttaaggtcggcgagcaagaccaggtaacgagcaaccaatacatctgtcctttgacccggcatgtcctgctgtacaggtccgcattagatcagaagtgcggttccatgacgagccacgttccctacaacgaagcgtaaactagtacccttctacacaggcaccgcccggagtaggaaggattatgcttttgcctttaggaatttctagattctggtccgtgctgcggcctgcaacgtggacttacttataactgcggttaggacgattcatctgaaggaatacgctctttttcgactgcagctcgcgtgacgcttggctgaaaaattgaaactggagcttcctctacggatcaacgtttaactacccactgcctattcctatgtactgatcgtcgagtcttgccaggattccgtcgcgggactcgtatcaacgttcagttgagttgtgtcatgctaaccgcacattgtgagtcaccaagtgtcccatttggtcaactgatctcgcaaaaggtaagggccgtagcaaagtcgccagcttcgtcaattgatggcctatttttaactcgccgcttacggtcggaatctgaacggagacgtctttggcagaatggcgttacgcaccaatctataaaaagtttttgttggaaaggaggataatttctactggaccggtgttgcgacggaggagatcgaattgctaatcaaccggtatgcacacttccattgctgtgcagttgccctaatcagctatccatccaacataaaactgtctgagtgcttaaacggtcacaccaaatgattgtggtgccgtatctatagaatatccttagagcgtctgcttcctcgtcacacgagaccggttagtcccaagcacaataacgaatccagcttctgtttgccttagctccggtgatgcatgtttctgcttccggcggtgcggatgccacagctgccactgcaggtggaggagaagctgccaagtccaaaccaactacatttactccaccagattccacccatatcgacttacttccaatagctggttttcgcgttgacccacattacaatgtatctacgactcaagttattttaatgtatagcgttctgtattcgacccttccataatgccctctatgtgaaactaacaacaatttgaccctcaaactttaagtataccagcttatggcaacagtctgcgagccatggaagggatataacctgacgcagattattgcactgtccaagatccttcatgccacatcttcaggagggcgggtgattagcaccgtaaacagcgggttgatatcatcaagcgaactgcagagaaatccgcgggaacactgggcttagcgcccatctcacccttaaaaattaaacgcatctcccggtttcaggcattgctaccctgcgccggctagcgccttcccaatcctgtggcttaagtctactgcgaaacaggttttataacagttccaccgcaatcaggtggccatttgtcctcactctaatcccatccacccgttgatagtcaaagattcctctaataggcccatgaacgtgcaaagttcccaatcgaacccacttggcacatacagtatccggcaaatgttatatcaacaagtcgcctgaacgtgccgcaacaacggatcaactgtagcttcgtgctgcctcagatgcatggctcgtgcccttctgttcgtgctgcatcggttgtatctcaatgactggactcagcatcgtagctaagtaggcagggattttagtggtgtctaaagaataccggcggtaggcccaaaaaattacctcgttcacccactaaagagatatcccgtacatctactatctactcaggaagatcaccactctagcgtgggagccgctataatggatgcaggcagcccgggttagcgtgatgaaggacgttttaagttactactactggagttgcgggcgcaagacgatggctaagtaagagcccagagtttaggccttgtctaaaccgtaatgaaactgacatcggtagtcaatgtgtcgacgagttttgatttcagtatatacgtactgttaaccgacgtctggatgtcagaaatttcgtgcatgtggcaggctcggttcgaggaatctcgttccggaagttagggtatcggcgagggaactagtataaggactcgactgatgcatggctcagctaacagcgggcactctatgtcctaaggatagtaagaggagcaggacaaccatccgggtgtaacgggttgatgcaagcgacatactaacaatgcctaggatagctgtgccatcaggcggaaaagaatccaatatgatggtgctcaggactcattattacaatatagtacatttaccagagaggtcccgcgggtcgccgaacacctacgcgaccctataagtttcttacactatcgataatggagaaagcttatttgagggactacgatcttttacacccatggactttcagccgagatatcaaaatcgtagttatgttgtagcctgtagatttgtattcacgggctgtactttagccgaggacagacccatatctgcttataataggtcatcatccctacattgtgtgagccagtctccaccgctcgatgccaggcagtccaatgttatgtaaaacgaaggcgaaagggtgtctaacaccatctgatcgatacaaactcgcactggccgcccacaaacgtcatggaaaggtagaaaattcgatgggctgacgctattaccgtatcataggtcgactcacgtgggtacgtgccgtagctcccatgtttaccgcttatgcgggtccagtggaaagagctttactggacgaataacgctgctgctttttaatccatatatgatactgcctagaacaagtacgggaaaatttcgacggcgtgcaatgtgcaatatgtttccgctatttgatcactcttggccgagtgcaatctctcactcgcgcttttgggctaacgacatagactcaatatcttagagtgagacgtgcggtctttcagtggagaaagccctgtttagaccacaggttcctattattcggatgaacgacctttaagataggtcaaccattatgacagttgcctgagtaagaacacgagcggagtatctgattctgatgcttagacgctgtcgcatcccgtgaaagctcatccagaccgggtgagcgtagacctataactacgccaacctacccggccgggaaatcatgtaggcaactcaacccgctcgcatgtaagttgtccataatatgaatttacccatccgaattgtatcgtggagttgttcggctagtggcaggagttcttagactaatgacaccctcactgttgcggcggtaacaaccattaactattacaagttgcggtttggtaaggttagggtaactgtagttaaaagtatttctgcgattgctcgtttcgtcagatcacttcacagcgcagtctacggcactagggacaagatttgtgtactgtggacccgtagccgagaaatccacggcattcatgagacgttactcgggaactattcagtcagtgtatgtagtcggcaaccggtagtggttccggaacaagcttttgaaaatcagttaatgtgggttgagctgctcagaggcgcccagttgtaccggaaaagtatgcatatctttaatggctatgcaagtatccaactatgctggcggcagggtagccatatccatcgggtccagaaggttcccattaaatacccgacactactagctatgttttgggtcctgttcccgagttacctgcataaggtgaataagccttagtaagttactgctctatgacattggctcagtcagactgttgcatacttatcttatagattcctatgcggaaaattccgaccgcagacttaataacttgatctaatgcccggtttctcagtacgatactagttaaaccggccagcacaggttcgtatatatccctgtatggatatgatctcgggtcgactttaagcgactaagtgctctagcgcctgatggcgtgctttcttctcccacgttgctatatcaccgagtgagtacccgcggatagagttgcttcgcaggtactcaaccacagttaggcaagtgcgaaggtattactgattaggcgtggccgcggccgtacacctcgttagtttgagggaagctgttccgatgactggctagcaggcctgggtgagtactagtgatcagcaaagcctctctgtggtcgcataggtccgacaatatgagcgcagtatgcgagtcccaccgaaattttgcaacgatcttgattctccctccagagtctaaatttcctatcctccaagtgtaactccagcatcagatgtttcttagaagtactcggagaatacaactttgagtacaatttgcgcgtccgtccggtttcccggtgggatttaccaacttaaaacttctagaccattattcacagcctagcgctgcgattggcggctcatgtgcggctcggctggtagggttggtgatgtggctaaccgtcgaagcgttgtaggacaccttatttggagaagagacgttgtgacgggaaaagtgtctcatgcgtaatgttccggagtatgggttcctgtgacgtgctgagagcagcactgtgcaagactggaaaggtggtgaagcaatacaccatgaatcccggatcgctgacgcgccctgtagcggcgcattaagcgcggcgggtgtggtggttacgcgcagcgtgaccgctacacttgccagcgccctagcgcccgctcctttcgctttcttcccttcctttctcgccacgttcgccggctttccccgtcaagctctaaatcgggggctccctttagggttccgatttagtgctttacggcacctcgaccccaaaaaacttgatttgggtgatggttcacgtagtgggccatcgccctgatagacggtttttcgccctttgacgttggagtccacgttcttt |
| 10,080-nt ssDNA sequence for length test | aatagtggactcttgttccaaactggaacaacactcaaccctatctcgggctattcttttgatttataagggattttgccgatttcggaacgggtacctacgaagagttccagcagggattccaagaaatggccaatgaagattgtttcccgcacctgggctctttcgcgccaatcgacgttaaacacttgaatttaagatgagcatcgcagaagtggatgatatgcgcgcctacggtccaatcggctgggtgagcgtccctataatctcacggataccgacgaaagtagggcaataatcgcgctgcatgagaacggatagcccggatgcaaaggtggaacgtaattgttaagagaagcaaatacgttaagctatcatttcccgtaaatcttttacatacgtaaggtggaagtctaaactagcctagctgcacaagcatcggactgcttgccctctatctttgtggaagttcaaggtaatggaagcggtagaacgtatgatcgtcacgcagacctaaagaatcacgccgcgcttacgtccaaccttgggacctacatctgtacagacccaggtccaggccgaggttatggatgtagatttgacttggctgatctacagtctcgagttacaatggcgtcactgctacctccatatgaaggataagaggcagccacaactcagccaccatcctaccggaaacgctttaagggcgaacgtgaagggaagtgcctacacgaccctctatgtgggaagtagtctacgatacacgatgattggaagtgtggcggcataggatgcatctacggaccgacactagttatagaagggtgcagtccttgacgcatacgaatagcgttagctcgcgatttcccgctgggttactcatggagagcggctcagacgcctagcccatactgaccgtgtaccaacgatccaccaattattttcagaacagcccttttatggcgaagggaaaacagagccttagtatgttacagtccgctctattgggagatctcgggcaggcggaagatattaattagaaccgcagaccgcatgatgtgccgtcctcaaatggcctggatatcccgtcacacgaattatctcagttgaagtgatcctttagggcccgagagcaccacaagctcccaaccgctgcgaagagtgctaacgagttgtcctatgttaagttattcgtatttaacccacgcccgcaccacctccgggaataatgtccaagatcatatcaaggaaacccagggcacgataagcggcacacagacaaccactttggctagttaagataacggatactctgacgtacagccggccattcgttctacttttgtgtagtcagatggtccgtaacaatggcccggctctgacaacaagtataagactgcatgacgggtgggtccacgttagatgttcgtgaggtagcccatacatcattctttacgaacaagcctctcccgagccagcagaggaggaatcagtgatcgttcacatggcgtacaacaaggcgtgcggcgccgtcggctagcgctgtgaacgtcgcagcgttgattccaaccctctgcccacgacttcgccgctaactggaagcggtattaaacccgaaggttccacgggtggccacttggtgaaggcggcatcttaaagttctgacctcaattactatggtgccgtcatctaaccaaccattaagaagtagaattgtaaccggttatcggaatcagtgtcgacatactcaatgcccttaccctccgtaactctatgttttccgggttgttcatatcggacatgctatcgcttagacgactcgtctatatagggcgttagtatatcatgggcgtactatcccggtcaatcgtctacgtcgcaaatcacctccctttgggcaggagagcacttctacactgcctggcgctggcaccgcacaatcaataatgagattgtccggtgagacttacaatcccatcagcgagtctgcaatcccgctgtcacgataacatagtactcgccttctggcggttcctcagctatcctaacgtctatctaaactcaatgtggcgttatttggttcaagcaggcgggtcgtcaacgcgaaacgtaagtcccacacggccgtaacgcaatgtggcaattccagagttaccgatcgaggccaatggcccggccaagccgcaaccgcatcctccgccggtaaaatgactaaatgagtggaaccgtcgcgcttacgctctcggtgcccggaaaactctacagcatattgtctcattggtcctttggcgtatacagacacttagactaactctgactgtcgagtaaattccaatgaacgccagcactcagtacgcacggtgcgtccagggatggttacaaagctagagatgtatgtgctctcatcgaataacccgtagtatttggacctgaataccctggaatccgagggtgagaccattttacgttatcactgtgacatgctttttccggtcaatacacgggaaactacggacgctatggtcgggataaacgccgcatcatgtcaagctgaggccgctaaacaaagcatcaaggtagatttatttacaagcaaggccttgcgggcccagtccatgcgctgctgagattacagggattccgtgccagcccttcaacgtctcaagacaactaacaggccttgaattcggccacactcaccggtcccacaatgtgccgggttcgcatcaccgctgcttgggatagtatgcacacaaaagtagcttccacgagcggttgcccaattagatggcgacccgcgtacaggctctaggaggctggaaagtccctctcaccgtcaaatatctgagacgttatacccgacccatcatacgcgaataaagtactagtctcgcctgtcgccctcaggtttaccaccaataggacacggaaggcgctctgacaccagccaactagacagacatcggcgaggtgtacttgacgcactttagaagctgctcccttgtgggaaccattgggcaaccgaacatagccgcaatccagtcgcatcatcggtgggtcactgaccgaggattttggcggctcacgccttctgggctagcttcttctggcttgttagttattgcgtttacgttactctatatcccactaactatctatatacctgtgctttcactacaatggctgcacagttatcttattttagcaaagctttgggttgcctggagtttcccaaagcgggacttgtaagcccgtcctatatcaggaggcggacgagagacgcagcatgttatcttctaatctgcgaacggtacccggtctttcgtcggctaactagatctgtgtcctaggtattcaaatcgggcgaagtcggtatcgaagaaaagccctttaacgtaaactgtattcgtcggccgccgttagctcaatgatataactctcatcgcgtgagtcatggcgccacattttataaaacacgctactatccaatcgaggagatcgctgcaccaataacagtctcaatccaacgaactagtatttcatcaccgatccgcgaatcgtgagaccgacccatcgtcttatcgtcctaagcagcacgggccacgacgtgcgaggcggcgtaacttgcgagttctacctatatacttgacaatctcagctactccaaactgcccatggttggttggccatatgtacaccgagtcctagtacatcctcactggacacgcgttcgcttgttggagagatgaaatccaagatattccttgtagggagctaatcttgccaacactcaaattcctgatgcctcccaaaatacccgggctcaggtcaaaaaagccatgaagcttcaagcccatgcttttcttgagtgattatcgctggccgggcgtataagttaatccagctaactggcgtgtcaacgaaagggtgggacacaatggttttccggctgtctcccagcaagtgtcagaggcatttgcctttctctcaaccaaagcgctacactacacaggtcatcccgtgaacattaggtagagttctccagtcagtcctcgacacgagtccagccactcgaacttagtttaaggtcggcgagcaagaccaggtaacgagcaaccaatacatctgtcctttgacccggcatgtcctgctgtacaggtccgcattagatcagaagtgcggttccatgacgagccacgttccctacaacgaagcgtaaactagtacccttctacacaggcaccgcccggagtaggaaggattatgcttttgcctttaggaatttctagattctggtccgtgctgcggcctgcaacgtggacttacttataactgcggttaggacgattcatctgaaggaatacgctctttttcgactgcagctcgcgtgacgcttggctgaaaaattgaaactggagcttcctctacggatcaacgtttaactacccactgcctattcctatgtactgatcgtcgagtcttgccaggattccgtcgcgggactcgtatcaacgttcagttgagttgtgtcatgctaaccgcacattgtgagtcaccaagtgtcccatttggtcaactgatctcgcaaaaggtaagggccgtagcaaagtcgccagcttcgtcaattgatggcctatttttaactcgccgcttacggtcggaatctgaacggagacgtctttggcagaatggcgttacgcaccaatctataaaaagtttttgttggaaaggaggataatttctactggaccggtgttgcgacggaggagatcgaattgctaatcaaccggtatgcacacttccattgctgtgcagttgccctaatcagctatccatccaacataaaactgtctgagtgcttaaacggtcacaccaaatgattgtggtgccgtatctatagaatatccttagagcgtctgcttcctcgtcacacgagaccggttagtcccaagcacaataacgaatccagcttctgtttgccttagctccggtgatgcatgtttctgcttccggcggtgcggatgccacagctgccactgcaggtggaggagaagctgccaagtccaaaccaactacatttactccaccagattccacccatatcgacttacttccaatagctggttttcgcgttgacccacattacaatgtatctacgactcaagttattttaatgtatagcgttctgtattcgacccttccataatgccctctatgtgaaactaacaacaatttgaccctcaaactttaagtataccagcttatggcaacagtctgcgagccatggaagggatataacctgacgcagattattgcactgtccaagatccttcatgccacatcttcaggagggcgggtgattagcaccgtaaacagcgggttgatatcatcaagcgaactgcagagaaatccgcgggaacactgggcttagcgcccatctcacccttaaaaattaaacgcatctcccggtttcaggcattgctaccctgcgccggctagcgccttcccaatcctgtggcttaagtctactgcgaaacaggttttataacagttccaccgcaatcaggtggccatttgtcctcactctaatcccatccacccgttgatagtcaaagattcctctaataggcccatgaacgtgcaaagttcccaatcgaacccacttggcacatacagtatccggcaaatgttatatcaacaagtcgcctgaacgtgccgcaacaacggatcaactgtagcttcgtgctgcctcagatgcatggctcgtgcccttctgttcgtgctgcatcggttgtatctcaatgactggactcagcatcgtagctaagtaggcagggattttagtggtgtctaaagaataccggcggtaggcccaaaaaattacctcgttcacccactaaagagatatcccgtacatctactatctactcaggaagatcaccactctagcgtgggagccgctataatggatgcaggcagcccgggttagcgtgatgaaggacgttttaagttactactactggagttgcgggcgcaagacgatggctaagtaagagcccagagtttaggccttgtctaaaccgtaatgaaactgacatcggtagtcaatgtgtcgacgagttttgatttcagtatatacgtactgttaaccgacgtctggatgtcagaaatttcgtgcatgtggcaggctcggttcgaggaatctcgttccggaagttagggtatcggcgagggaactagtataaggactcgactgatgcatggctcagctaacagcgggcactctatgtcctaaggatagtaagaggagcaggacaaccatccgggtgtaacgggttgatgcaagcgacatactaacaatgcctaggatagctgtgccatcaggcggaaaagaatccaatatgatggtgctcaggactcattattacaatatagtacatttaccagagaggtcccgcgggtcgccgaacacctacgcgaccctataagtttcttacactatcgataatggagaaagcttatttgagggactacgatcttttacacccatggactttcagccgagatatcaaaatcgtagttatgttgtagcctgtagatttgtattcacgggctgtactttagccgaggacagacccatatctgcttataataggtcatcatccctacattgtgtgagccagtctccaccgctcgatgccaggcagtccaatgttatgtaaaacgaaggcgaaagggtgtctaacaccatctgatcgatacaaactcgcactggccgcccacaaacgtcatggaaaggtagaaaattcgatgggctgacgctattaccgtatcataggtcgactcacgtgggtacgtgccgtagctcccatgtttaccgcttatgcgggtccagtggaaagagctttactggacgaataacgctgctgctttttaatccatatatgatactgcctagaacaagtacgggaaaatttcgacggcgtgcaatgtgcaatatgtttccgctatttgatcactcttggccgagtgcaatctctcactcgcgcttttgggctaacgacatagactcaatatcttagagtgagacgtgcggtctttcagtggagaaagccctgtttagaccacaggttcctattattcggatgaacgacctttaagataggtcaaccattatgacagttgcctgagtaagaacacgagcggagtatctgattctgatgcttagacgctgtcgcatcccgtgaaagctcatccagaccgggtgagcgtagacctataactacgccaacctacccggccgggaaatcatgtaggcaactcaacccgctcgcatgtaagttgtccataatatgaatttacccatccgaattgtatcgtggagttgttcggctagtggcaggagttcttagactaatgacaccctcactgttgcggcggtaacaaccattaactattacaagttgcggtttggtaaggttagggtaactgtagttaaaagtatttctgcgattgctcgtttcgtcagatcacttcacagcgcagtctacggcactagggacaagatttgtgtactgtggacccgtagccgagaaatccacggcattcatgagacgttactcgggaactattcagtcagtgtatgtagtcggcaaccggtagtggttccggaacaagcttttgaaaatcagttaatgtgggttgagctgctcagaggcgcccagttgtaccggaaaagtatgcatatctttaatggctatgcaagtatccaactatgctggcggcagggtagccatatccatcgggtccagaaggttcccattaaatacccgacactactagctatgttttgggtcctgttcccgagttacctgcataaggtgaataagccttagtaagttactgctctatgacattggctcagtcagactgttgcatacttatcttatagattcctatgcggaaaattccgaccgcagacttaataacttgatctaatgcccggtttctcagtacgatactagttaaaccggccagcacaggttcgtatatatccctgtatggatatgatctcgggtcgactttaagcgactaagtgctctagcgcctgatggcgtgctttcttctcccacgttgctatatcaccgagtgagtacccgcggatagagttgcttcgcaggtactcaaccacagttaggcaagtgcgaaggtattactgattaggcgtggccgcggccgtacacctcgttagtttgagggaagctgttccgatgactggctagcaggcctgggtgagtactagtgatcagcaaagcctctctgtggtcgcataggtccgacaatatgagcgcagtatgcgagtcccaccgaaattttgcaacgatcttgattctccctccagagtctaaatttcctatcctccaagtgtaactccagcatcagatgtttcttagaagtactcggagaatacaactttgagtacaatttgcgcgtccgtccggtttcccggtgggatttaccaacttaaaacttctagaccattattcacagcctagcgctgcgattggcggctcatgtgcggctcggctggtagggttggtgatgtggctaaccgtcgaagcgttgtaggacaccttatttggagaagagacgttgtgacgggaaaagtgtctcatgcgtaatgttccggagtatgggttcctgtgacgtgtagcagccttcaaatggacggatttacgccaatatttcattaagtgagcctaaaattagcccgcaacgaggtgatctgggagactacaatctaaggttgcgagtgtgatcagctcagaaacaatagctcgtgaacctcgattcagcagggattgcactctgtggtccgtttctccactctctggttggtttacgagtgagccctttaaccctggtaggatccataaaacatgtaaaaccaccttgcttgtgctagcattagggaccggtgttcacacccatctatatggaggctcctggtgcacctcggacaaatgaggctttaagctatcgattccgaatggctacctggcgcatgcaaggtgccctgttcatgtccagtcagtgctgatgggaccgctgcgagagacctagtagattcttcgcataaaaatatatgtgttaacacggtactgtccgtggtatgccgttagaaataggtgcccatccgactcacgtcctgccagcgaccaatggaatcccgctaaacagaaatgattatagtgagcaaggcatagtaaatatcagtccggtactactctacttcgagtccctaaagaacgacaaggatctgtgaataaaactatagatcaattcagtgcttaccctcctatctgcttaaggtgtaattgtatgagggtgccgcgaccttcgtcatttcgaaaatgcccagttgacctaaaaccaggcaatgtctagaaccgcatagggacttaatgacaggccgccgatccagcaatgatttattcgccgaatagtcccaagtgttcacgggagatcgttattgggaggcgggtgtatgtgtgagcgccctacacccttctatcttctcgtacggccttgttgagtatttatctgccgacttgatcaaaatagggtccgccatcgtcacattacgacttcgaaatcccgatgtgctgcttagaaattttgtaacatcggtaccaagagagtgcaaaacgaataattgctgagagcagcactgtgcaagactggaaaggtggtgaagcaatacaccatgaatcccggatcgctgacgcgccctgtagcggcgcattaagcgcggcgggtgtggtggttacgcgcagcgtgaccgctacacttgccagcgccctagcgcccgctcctttcgctttcttcccttcctttctcgccacgttcgccggctttccccgtcaagctctaaatcgggggctccctttagggttccgatttagtgctttacggcacctcgaccccaaaaaacttgatttgggtgatggttcacgtagtgggccatcgccctgatagacggtttttcgccctttgacgttggagtccacgttcttt |
| 20,724-nt ssDNA sequence for length test | aatagtggactcttgttccaaactggaacaacactcaaccctatctcgggctattcttttgatttataagggattttgccgatttcggaacgggtacctacgaagagttccagcagggattccaagaaatggccaatgaagattgtttcccgcacctgggctctttcgcgccaatcgacgttaaacacttgaatttaagatgagcatcgcagaagtggatgatatgcgcgcctacggtccaatcggctgggtgagcgtccctataatctcacggataccgacgaaagtagggcaataatcgcgctgcatgagaacggatagcccggatgcaaaggtggaacgtaattgttaagagaagcaaatacgttaagctatcatttcccgtaaatcttttacatacgtaaggtggaagtctaaactagcctagctgcacaagcatcggactgcttgccctctatctttgtggaagttcaaggtaatggaagcggtagaacgtatgatcgtcacgcagacctaaagaatcacgccgcgcttacgtccaaccttgggacctacatctgtacagacccaggtccaggccgaggttatggatgtagatttgacttggctgatctacagtctcgagttacaatggcgtcactgctacctccatatgaaggataagaggcagccacaactcagccaccatcctaccggaaacgctttaagggcgaacgtgaagggaagtgcctacacgaccctctatgtgggaagtagtctacgatacacgatgattggaagtgtggcggcataggatgcatctacggaccgacactagttatagaagggtgcagtccttgacgcatacgaatagcgttagctcgcgatttcccgctgggttactcatggagagcggctcagacgcctagcccatactgaccgtgtaccaacgatccaccaattattttcagaacagcccttttatggcgaagggaaaacagagccttagtatgttacagtccgctctattgggagatctcgggcaggcggaagatattaattagaaccgcagaccgcatgatgtgccgtcctcaaatggcctggatatcccgtcacacgaattatctcagttgaagtgatcctttagggcccgagagcaccacaagctcccaaccgctgcgaagagtgctaacgagttgtcctatgttaagttattcgtatttaacccacgcccgcaccacctccgggaataatgtccaagatcatatcaaggaaacccagggcacgataagcggcacacagacaaccactttggctagttaagataacggatactctgacgtacagccggccattcgttctacttttgtgtagtcagatggtccgtaacaatggcccggctctgacaacaagtataagactgcatgacgggtgggtccacgttagatgttcgtgaggtagcccatacatcattctttacgaacaagcctctcccgagccagcagaggaggaatcagtgatcgttcacatggcgtacaacaaggcgtgcggcgccgtcggctagcgctgtgaacgtcgcagcgttgattccaaccctctgcccacgacttcgccgctaactggaagcggtattaaacccgaaggttccacgggtggccacttggtgaaggcggcatcttaaagttctgacctcaattactatggtgccgtcatctaaccaaccattaagaagtagaattgtaaccggttatcggaatcagtgtcgacatactcaatgcccttaccctccgtaactctatgttttccgggttgttcatatcggacatgctatcgcttagacgactcgtctatatagggcgttagtatatcatgggcgtactatcccggtcaatcgtctacgtcgcaaatcacctccctttgggcaggagagcacttctacactgcctggcgctggcaccgcacaatcaataatgagattgtccggtgagacttacaatcccatcagcgagtctgcaatcccgctgtcacgataacatagtactcgccttctggcggttcctcagctatcctaacgtctatctaaactcaatgtggcgttatttggttcaagcaggcgggtcgtcaacgcgaaacgtaagtcccacacggccgtaacgcaatgtggcaattccagagttaccgatcgaggccaatggcccggccaagccgcaaccgcatcctccgccggtaaaatgactaaatgagtggaaccgtcgcgcttacgctctcggtgcccggaaaactctacagcatattgtctcattggtcctttggcgtatacagacacttagactaactctgactgtcgagtaaattccaatgaacgccagcactcagtacgcacggtgcgtccagggatggttacaaagctagagatgtatgtgctctcatcgaataacccgtagtatttggacctgaataccctggaatccgagggtgagaccattttacgttatcactgtgacatgctttttccggtcaatacacgggaaactacggacgctatggtcgggataaacgccgcatcatgtcaagctgaggccgctaaacaaagcatcaaggtagatttatttacaagcaaggccttgcgggcccagtccatgcgctgctgagattacagggattccgtgccagcccttcaacgtctcaagacaactaacaggccttgaattcggccacactcaccggtcccacaatgtgccgggttcgcatcaccgctgcttgggatagtatgcacacaaaagtagcttccacgagcggttgcccaattagatggcgacccgcgtacaggctctaggaggctggaaagtccctctcaccgtcaaatatctgagacgttatacccgacccatcatacgcgaataaagtactagtctcgcctgtcgccctcaggtttaccaccaataggacacggaaggcgctctgacaccagccaactagacagacatcggcgaggtgtacttgacgcactttagaagctgctcccttgtgggaaccattgggcaaccgaacatagccgcaatccagtcgcatcatcggtgggtcactgaccgaggattttggcggctcacgccttctgggctagcttcttctggcttgttagttattgcgtttacgttactctatatcccactaactatctatatacctgtgctttcactacaatggctgcacagttatcttattttagcaaagctttgggttgcctggagtttcccaaagcgggacttgtaagcccgtcctatatcaggaggcggacgagagacgcagcatgttatcttctaatctgcgaacggtacccggtctttcgtcggctaactagatctgtgtcctaggtattcaaatcgggcgaagtcggtatcgaagaaaagccctttaacgtaaactgtattcgtcggccgccgttagctcaatgatataactctcatcgcgtgagtcatggcgccacattttataaaacacgctactatccaatcgaggagatcgctgcaccaataacagtctcaatccaacgaactagtatttcatcaccgatccgcgaatcgtgagaccgacccatcgtcttatcgtcctaagcagcacgggccacgacgtgcgaggcggcgtaacttgcgagttctacctatatacttgacaatctcagctactccaaactgcccatggttggttggccatatgtacaccgagtcctagtacatcctcactggacacgcgttcgcttgttggagagatgaaatccaagatattccttgtagggagctaatcttgccaacactcaaattcctgatgcctcccaaaatacccgggctcaggtcaaaaaagccatgaagcttcaagcccatgcttttcttgagtgattatcgctggccgggcgtataagttaatccagctaactggcgtgtcaacgaaagggtgggacacaatggttttccggctgtctcccagcaagtgtcagaggcatttgcctttctctcaaccaaagcgctacactacacaggtcatcccgtgaacattaggtagagttctccagtcagtcctcgacacgagtccagccactcgaacttagtttaaggtcggcgagcaagaccaggtaacgagcaaccaatacatctgtcctttgacccggcatgtcctgctgtacaggtccgcattagatcagaagtgcggttccatgacgagccacgttccctacaacgaagcgtaaactagtacccttctacacaggcaccgcccggagtaggaaggattatgcttttgcctttaggaatttctagattctggtccgtgctgcggcctgcaacgtggacttacttataactgcggttaggacgattcatctgaaggaatacgctctttttcgactgcagctcgcgtgacgcttggctgaaaaattgaaactggagcttcctctacggatcaacgtttaactacccactgcctattcctatgtactgatcgtcgagtcttgccaggattccgtcgcgggactcgtatcaacgttcagttgagttgtgtcatgctaaccgcacattgtgagtcaccaagtgtcccatttggtcaactgatctcgcaaaaggtaagggccgtagcaaagtcgccagcttcgtcaattgatggcctatttttaactcgccgcttacggtcggaatctgaacggagacgtctttggcagaatggcgttacgcaccaatctataaaaagtttttgttggaaaggaggataatttctactggaccggtgttgcgacggaggagatcgaattgctaatcaaccggtatgcacacttccattgctgtgcagttgccctaatcagctatccatccaacataaaactgtctgagtgcttaaacggtcacaccaaatgattgtggtgccgtatctatagaatatccttagagcgtctgcttcctcgtcacacgagaccggttagtcccaagcacaataacgaatccagcttctgtttgccttagctccggtgatgcatgtttctgcttccggcggtgcggatgccacagctgccactgcaggtggaggagaagctgccaagtccaaaccaactacatttactccaccagattccacccatatcgacttacttccaatagctggttttcgcgttgacccacattacaatgtatctacgactcaagttattttaatgtatagcgttctgtattcgacccttccataatgccctctatgtgaaactaacaacaatttgaccctcaaactttaagtataccagcttatggcaacagtctgcgagccatggaagggatataacctgacgcagattattgcactgtccaagatccttcatgccacatcttcaggagggcgggtgattagcaccgtaaacagcgggttgatatcatcaagcgaactgcagagaaatccgcgggaacactgggcttagcgcccatctcacccttaaaaattaaacgcatctcccggtttcaggcattgctaccctgcgccggctagcgccttcccaatcctgtggcttaagtctactgcgaaacaggttttataacagttccaccgcaatcaggtggccatttgtcctcactctaatcccatccacccgttgatagtcaaagattcctctaataggcccatgaacgtgcaaagttcccaatcgaacccacttggcacatacagtatccggcaaatgttatatcaacaagtcgcctgaacgtgccgcaacaacggatcaactgtagcttcgtgctgcctcagatgcatggctcgtgcccttctgttcgtgctgcatcggttgtatctcaatgactggactcagcatcgtagctaagtaggcagggattttagtggtgtctaaagaataccggcggtaggcccaaaaaattacctcgttcacccactaaagagatatcccgtacatctactatctactcaggaagatcaccactctagcgtgggagccgctataatggatgcaggcagcccgggttagcgtgatgaaggacgttttaagttactactactggagttgcgggcgcaagacgatggctaagtaagagcccagagtttaggccttgtctaaaccgtaatgaaactgacatcggtagtcaatgtgtcgacgagttttgatttcagtatatacgtactgttaaccgacgtctggatgtcagaaatttcgtgcatgtggcaggctcggttcgaggaatctcgttccggaagttagggtatcggcgagggaactagtataaggactcgactgatgcatggctcagctaacagcgggcactctatgtcctaaggatagtaagaggagcaggacaaccatccgggtgtaacgggttgatgcaagcgacatactaacaatgcctaggatagctgtgccatcaggcggaaaagaatccaatatgatggtgctcaggactcattattacaatatagtacatttaccagagaggtcccgcgggtcgccgaacacctacgcgaccctataagtttcttacactatcgataatggagaaagcttatttgagggactacgatcttttacacccatggactttcagccgagatatcaaaatcgtagttatgttgtagcctgtagatttgtattcacgggctgtactttagccgaggacagacccatatctgcttataataggtcatcatccctacattgtgtgagccagtctccaccgcgaaagtgaaacgtgatttcatgcgtcattttgaacattttgtaaatcttatttaataatgtgtgcggcaattcacatttaatttatgaatgttttcttaacatcgcggcaactcaagaaacggcaggttcggatcttagctactagagaaagaggagaaatactagaatgatggcttcctccgaggatgttatcaaagagttcatgcgtttcaaagttcgtatggaaggttccgttaacggtcacgagttcgaaatcgaaggtgaaggtgaaggtcgtccgtacgaaggtacccagaccgctaaactgaaagttaccaaaggtggtccgctgccgttcgcttgggacatcctgtccccgcagttccagtacggttccaaagcttacgttaaacacccggctgacatcccggactacctgaaactgtccttcccggaaggtttcaaatgggaacgtgttatgaacttcgaagatggtggtgttgttaccgttacccaggactcctccctgcaagacggtgagttcatctacaaagttaaactgcgtggtaccaacttcccgtccgacggtccggttatgcagaaaaaaaccatgggttgggaagcttccaccgaacgtatgtacccggaggatggtgctctgaaaggtgaaatcaaaatgcgtctgaaactgaaagacggtggtcactacgacgctgaagttaaaaccacctacatggctaaaaaaccggttcagctgccgggtgcttacaaaaccgacatcaaactggacatcacctcccacaacgaggactacaccatcgttgaacagtacgaacgtgctgaaggtcgtcactccaccggtgcttaataaaggtccaggcatcaaataaaacgaaaggctcagtcgaaagactgggcctttcgttttatctgttgtttgtcggtgaacgctctctactagagtcacactggctcaccttcgggtgggcctttctgcgtttataggttctGACGATTTGGAAGTGACACGCAAGAAGCTGGTCGATGACTGTCACCACTTCCGCCTCGAAGAACCTAACTTCTCTCTCGCCTCCAGCATCAGCAAGGATATTGAGTCCTGTGCTCAGATTTGGGCCTTCTACGAAGAGTTCCAGCAGGGATTCCAAGAAATGGCCAATGAAGATTGGATCACCTTTCGCACTAAGACCTACTTGTTTGAGGAGTTTCTGATGAATTGGCACGACCGCCTCAGGAAAGTGGAGGAGCATTCTGTGATGACTGTCAAGCTCCAATCTGAGGTGGACAAATATAAGATTGTTATCCCTATCCTGAAGTACGTCCGCGGAGAACACCTGTCACCCGATCACTGGCTGGATCTGTTCCGCTTGCTGGGTCTGCCTCGCGGCACATCTCTGGAGAAACTGCTGTTCGGTGACCTGCTGAGAGTTGCCGATACCATCGTGGCCAAGGCTGCTGACCTGAAAGATCTGAACTCACGCGCCCAGGGTGAAGTGACCATCCGCGAAGCACTCAGGGAACTGGATTTGTGGGGCGTGGGTGCTGTGTTCACACTGATCGACTATGAGGACTCCCAGAGCCGCACCATGAAGCTGATCAAGGATTGGAAGGACATCGTCAACCAGGTGGGCGACAATAGATGCCTCCTGCAGTCCTTGAAGGACTCACCATACTATAAAGGCTTTGAAGACAAGGTCAGCATCTGGGAAAGGAAACTCGCCGAACTGGACGAATATTTGCAGAACCTCAACCATATTCAGAGAAAGTGGGTTTACCTCGAACCAATCTTTGGTCGCGGAGCCCTGCCCAAAGAGCAGACCAGATTCAACAGGGTGGATGAAGATTTCCGCAGCATCATGACAGATATCAAGAAGGACAATCGCGTCACAACCTTGACTACCCACGCAGGCATTCGCAACTCACTGCTGACCATCCTGGACCAATTGCAGAGATGCCAGCGCAGCCTCAACGAGTTCCTGGAGGAGAAGCGCAGCGCCTTCCCTCGCTTCTACTTCATCGGAGACGATGACCTGCTGGAGATCTTGGGCCAGTCAACCAATCCATCCGTGATTCAGTCTCACCTCAAGAAGCTGTTTGCTGGTATCAACTCTGTCTGTTTCGATGAGAAGTCTAAGCACATTACTGCAATGAAGTCCTTGGAGGGAGAAGTTGTGCCATTCAAGAATAAGGTGCCCTTGTCCAATAACGTCGAAACCTGGCTGAACGATCTGGCCCTGGAGATGAAGAAGACCCTGGAGCAGCTGCTGAAGGAGTGCGTGACAACCGGACGCAGCTCTCAGGGAGCTGTGGACCCTTCTCTGTTCCCATCACAGATCCTGTGCTTGGCCGAACAGATCAAGTTTACCGAAGATGTGGAGAACGCAATTAAAGATCACTCCCTGCACCAGATTGAGACACAGCTGGTGAACAAATTGGAGCAGTATACTAACATCGACACATCTTCCGAGGACCCAGGTAACACAGAGTCCGGTATTCTGGAGCTGAAACTGAAAGCACTGATTCTCGACATTATCCATAACATCGACGTGGTCAAGCAGCTGAACCAAATCCAAGTGCACACCACCGAAGATTGGGCCTGGAAGAAGCAGTTGAGGTTCTACATGAAGTCCGACCACACCTGTTGCGTTCAGATGGTTGACAGCGAGTTCCAGTACACCTATGAGTACCAAGGAAATGCCAGCAAGCTCGTTTACACTCCACTCACTGACAAGTGTTACCTCACCTTGACACAGGCTATGAAGATGGGCCTGGGAGGCAACCCATACGGTCCAGCTGGCACTGGTAAGACAGAGAGCGTTAAGGCACTCGGAGGTCTGCTGGGCAGGCAGGTCCTCGTGTTCAACTGTGATGAAGGAATCGACGTTAAGTCCATGGGAAGAATCTTTGTTGGCCTCGTTAAGTGTGGAGCTTGGGGTTGCTTCGACGAGTTCAACAGGCTGGAGGAATCTGTGCTGAGCGCCGTCTCTATGCAGATCCAGACCATCCAGGACGCATTGAAGAACCACAGGACCGTCTGCGAGCTGTTGGGTAAGGAAGTGGAGGTGAACTCCAACTCCGGAATCTTCATCACAATGAATCCCGCAGGTAAAGGATATGGAGGAAGACAGAAACTCCCAGACAACCTGAAGCAGCTGTTCCGCCCAGTGGCTATGTCCCATCCAGACAATGAGCTGATCGCCGAAGTCATCCTCTATTCCGAGGGATTCAAAGATGCTAAAGTTCTCTCCAGAAAGCTCGTGGCCATCTTCAATCTGTCAAGAGAACTCCTGACACCTCAGCAGCATTACGACTGGGGTCTGAGAGCCCTCAAGACCGTCCTGAGAGGTTCAGGAAATCTCCTCAGGCAGCTGAACAAGAGCGGTACAACACAGAATGCAAATGAGAGCCACATTGTCGTCCAGGCTCTGAGGCTGAATACCATGTCAAAGTTCACATTCACAGACTGCACAAGATTTGACGCTCTGATTAAAGATGTGTTCCCTGGTATTGAACTCAAAGAAGTGGAGTATGACGAGCTGAGCGCCGCTTTGAAGCAGGTGTTTGAGGAGGCTAACTATGAGATTATCCCTAATCAGATCAAGAAAGCATTGGAACTGTATGAACAGCTGTGTCAGAGGATGGGAGTGGTGATTGTGGGCCCATCAGGCGCAGGTAAGAGCACTCTCTGGAGAATGCTGAGAGCAGCACTGTGCAAGACTGGAAAGGTGGTGAAGCAATACACCATGAATCCCAAGGCCATGCCCAGGTACCAACTGCTGGGCCATATCGACATGGACACCAGAGAATGGAGCGACGGCGTGCTCACAAACTCCGCCAGACAAGTCGTGCGCGAACCTCAAGACGTCAGCTCTTGGATCATCTGCGATGGTGATATTGACCCTGAGTGGATCGAGTCCCTGAATTCCGTGTTGGATGACAACAGGCTCCTCACAATGCCTTCTGGTGAGAGAATCCAGTTCGGTCCTAACGTGAACTTCGTGTTCGAGACACACGATCTCAGCTGTGCTAGCCCAGCTACTATCTCCCGCATGGGAATGATCTTCTTGTCCGACGAGGAGACAGATTTGAACTCATTGATCAAGTCTTGGCTCAGAAACCAGCCTGCAGAATATAGGAATAACCTGGAGAACTGGATCGGTGATTACTTCGAGAAGGCTTTGCAGTGGGTGCTGAAACAGAACGACTATGTCGTCGAAACCAGCCTGGTCGGTACAGTTATGAACGGACTCTCCCATCTGCACGGATGCAGAGATCACGATGAGTTTATCATCAATTTGATCCGCGGACTGGGAGGTAACTTGAATATGAAATCTCGCCTGGAGTTCACTAAAGAAGTGTTCCACTGGGCTAGGGAGTCACCACCTGACTTCCACAAACCTATGGACACCTACTATGATTCCACAAGAGGCAGGTTGGCCACCTACGTGCTGAAGAAGCCTGAGGACCTCACCGCTGACGACTTCTCCAACGGACTGACTCTGCCCGTGATCCAGACTCCAGACATGCAGCGCGGACTCGATTACTTTAAGCCCTGGCTCAGCTCCGATACCAAGCAACCTTTCATTCTCGTGGGACCAGAGGGATGTGGTAAAGGAATGCTCCTGAGGTATGCATTCTCCCAGCTCCGCTCAACCCAAATTGCCACTGTTCACTGTTCAGCCCAAACAACTTCAAGGCATCTCCTCCAGAAGCTCAGCCAGACCTGTATGGTTATCAGCACCAACACCGGCAGAGTTTACCGCCCAAAGGATTGTGAGCGCCTCGTGTTGTACCTCAAAGATATCAATCTGCCCAAACTCGATAAGTGGGGCACTTCCACCCTCGTGGCATTTCTCCAACAGGTGCTGACCTACCAGGGCTTCTACGACGAGAACCTGGAGTGGGTCGGATTGGAGAACATCCAGATTGTGGCTTCCATGTCTGCCGGCGGTAGGTTGGGAAGGCATAAGCTCACCACCAGGTTTACATCAATTGTGAGACTGTGCTCAATCGATTATCCCGAGCGCGAACAGTTGCAGACCATCTATGGCGCCTACCTCGAACCTGTCCTCCACAAGAATTTGAAGAACCATAGCATCTGGGGCTCATCAAGCAAGATCTACCTCTTGGCTGGCTCTATGGTTCAGGTGTACGAACAAGTCCGCGCCAAGTTCACCGTCGATGATTACTCACATTACTTCTTCACACCCTGCATTCTGACACAATGGGTTCTGGGACTGTTTCGCTATGACCTGGAGGGAGGCTCATCAAACCACCCATTGGATTATGTCCTCGAAATCGTGGCTTACGAAGCCCGCAGGCTCTTTAGAGATAAGATTGTTGGCGCTAAGGAACTGCATCTGTTCGATATTATCCTCACCTCTGTGTTTCAAGGTGATTGGGGCTCTGATATCCTGGATAATATGTCCGATTCCTTCTATGTGACATGGGGAGCCAGGCACAACTCCGGTGCAAGGGCTGCTCCAGGCCAACCATTGCCACCTCATGGCAAGCCTCTCGGCAAACTGAACTCAACTGACTTGAAGGACGTGATCAAGAAGGGCCTGATTCACTACGGCCGCGACAACCAGAACCTGGACATTCTGTTGTTCCACGAGGTCCTGGAGTATATGTCCAGAATTGATCGCGTCCTGTCTTTCCCTGGTGGATCACTGCTGCTGGCCGGACGCTCTGGAGTTGGAAGACGCACTATTACTTCTCTGGTCAGCCATATGCATGGAGCCGTCCTGTTCTCTCCAAAGATCAGCAGAGGCTACGAACTCAAGCAATTCAAGAACGATCTGAAACACGTCTTGCAACTCGCCGGTATCGAGGCCCAGCAGGTCGTCCTCCTCTTGGAGGACTATCAATTCGTCCACCCAACCTTCCTGGAGATGATCAACTCCCTGCTGTCCTCTGGCGAGGTGCCCGGCTTGTACACTTTGGAGGAACTGGAGCCACTGCTCCTCCCATTGAAGGATCAGGCATCACAGGACGGCTTCTTCGGCCCAGTGTTCAATTACTTCACCTATCGCATTCAACAGAATCTCCACATTGTGCTGATTATGGACAGCGCTAATTCCAATTTCATGATCAATTGCGAGAGCAATCCCGCCCTCCATAAGAAGTGCCAGGTCCTCTGGATGGAGGGTTGGTCTAATTCTTCTATGAAGAAGATTCCCGAGATGTTGTTTAGCGAGACTGGAGGCGGTGAGAAGTACAACGACAAGAAGCGCAAAGAGGAGAAGAAGAAGAACTCCGTCGATCCTGATTTCCTCAAGAGCTTCCTGCTGATCCACGAGTCTTGCAAAGCTTACGGAGCTACTCCTAGCCAGTACATGACCTTCCTCCACGTCTATTCCGCCATCTCCAGCTCAAAGAAGAAGGAGCTGCTGAAGCGCCAATCTCATCTGCAGGCCGGAGTCAGCAAGCTGAACGAAGCCAAAGCTCTGGTGGATGAACTGAATCGCAAGGCTGGCGAACAATCAGTCCTCCTGAAGACTAAGCAGGATGAAGCTGACGCTGCCCTGCAAATGATTACCGTGTCTATGCAGGATGCTTCCGAGCAGAAGACAGAGCTGGAGAGGCTGAAGCACCGCATCGCAGAGGAGGTGGTCAAGATCGAGGAGAGAAAGAACAAGATTGACGACGAACTCAAAGAGGTGCAGCCTCTGGTGAACGAGGCCAAGCTCGCCGTGGGTAATATCAAACCAGAGTCTCTCTCAGAGATCAGGTCACTGAGAATGCCACCAGACGTTATCCGCGACATCCTGGAGGGCGTCCTGCGCTTGATGGGTATCTTTGACACCTCTTGGGTGTCTATGAAGTCTTTCTTGGCCAAGCGCGGTGTCAGGGAGGACATTGCTACTTTCGACGCTAGGAACATCTCCAAGGAAATTAGGGAATCTGTGGAGGAACTGCTGTTCAAGAATAAAGGTTCATTTGATCCCAAGAACGCTAAGAGAGCATCAACTGCAGCTGCACCCTTGGCAGCCTGGGTTAAGGCCAATATCCAGTACTCTCACGTGCTCGAACGCATCCACCCTCTGGAGACTGAGCAAGCCGGCCTGGAAAGCAACCTGAAGAAGACCGAGGATAGAAAGAGGAAACTGGAAGAACTCCTCAATTCTGTCGGTCAGAAGGTGTCAGAACTGAAGGAGAAATTTCAGAGCAGGACCTCAGAAGCAGCTAAGTTGGAAGCTGAGGTGTCCAAGGCTCAGGAGACTATCAAAGCAGCTGAAGTGTTGATTAATCAGCTGGACCGCGAACACAAGAGATGGAATGCTCAGGTCGTCGAGATTACTGAGGAGCTGGCAACCCTCCCAAAGAGGGCTCAGTTGGCCGCAGCCTTTATCACCTACTTGTCCGCTGCACCTGAGTCTCTCAGAAAGACATGTCTGGAGGAGTGGACCAAGTCTGCCGGCCTGGAGAAGTTTGACCTGAGAAGATTTCTCTGCACCGAGTCAGAGCAGCTGATCTGGAAGTCTGAAGGTCTGCCCAGCGATGACCTCTCAATCGAGAACGCACTGGTTATCTTGCAATCCCGCGTTTGCCCTTTCCTCATCGATCCCAGCTCACAGGCTACTGAGTGGCTGAAGACTCACTTGAAGGATTCCAGGCTGGAAGTGATCAACCAGCAAGACTCCAACTTCATCACTGCCCTGGAACTCGCCGTCCGCTTCGGCAAGACCTTGATCATCCAGGAGATGGATGGCGTGGAGCCAGTTCTGTACCCTCTGTTGCGCAGAGATTTGGTGGCCCAAGGCCCACGCTATGTGGTCCAGATTGGAGATAAGATCATCGACTATAACGAGGAGTTTCGCCTGTTCCTGAGCACTAGGAATCCCAACCCATTCATTCCACCAGACGCTGCCAGCATCGTCACAGAGGTTAATTTCACAACCACCCGCTCAGGACTGAGAGGCCAGCTCCTGGCCCTCACCATCCAACATGAGAAACCCGATTTGGAAGAACAGAAGACTAAGCTGCTCCAACAGGAGGAGGATAAGAAGATCCAACTGGCCAAACTCGAAGAATCTCTGCTGGAGACATTGGCTACTTCTCAGGGCAACATCCTGGAGAACAAGGACCTGATCGAGTCTCTGAATCAGACTAAAGCATCTTCCGCCCTCATCCAAGAGTCTCTGAAGGAATCTTATAAGCTGCAAATCTCTCTCGACCAGGAGCGCGACGCATATCTGCCACTCGCTGAGTCTGCTTCAAAGATGTACTTCATTATCTCTGACCTCTCCAAGATCAATAATATGTACAGGTTCTCCCTGGCCGCCTTTCTGAGGTTGTTTCAAAGGGCACTCCAGAATAAGCAGGATTCTGAGAACACAGAACAGAGGATTCAATCCCTGATCAGCTCCCTGCAGCACATGGTGTACGAATACATCTGCCGCTGCCTGTTCAAGGCCGACCAGCTGATGTTCGCCCTGCATTTCGTCAGAGGTATGCATCCAGAATTGTTCCAGGAGAACGAATGGGATACCTTCACTGGCGTGGTGGTCGGAGACATGTTGCGCAAAGCCGACTCCCAGCAGAAGATCAGGGACCAGTTGCCTTCATGGATCGATCAAGAGAGATCCTGGGCTGTGGCAACCTTGAAGATCGCTCTGCCTTCCCTGTACCAGACTCTGTGTTTCGAGGACGCCGCTTTGTGGCGCACCTACTACAACAACAGCATGTGTGAGCAGGAATTTCCTAGCATCTTGGCTAAGAAGGTCAGCTTGTTCCAGCAGATCCTCGTCGTGCAGGTGCTCAGGCCAGATAGATTGCAGTCAGCTATGGCCCTCTTTGCCTGCAAGACCTTGGGTTTGAAGGAAGTCTCTCCACTCCCACTCAATCTCAAGAGGCTGTACAAGGAGACACTCGAAATCGAGCCCATTCTGATCATTATTTCTCCAGGAGCCGATCCCTCCCAGGAACTCCAGGAGCTCGCCAACGCCGAAAGATCAGGAGAATGTTACCACCAGGTTGCAATGGGCCAGGGCCAAGCTGACTTGGCTATCCAAATGCTCAAGGAATGCGCAAGGAATGGTGACTGGCTGTGTTTGAAGAACTTGCACCTGGTTGTCTCCTGGCTGCCTGTCTTGGAGAAGGAACTGAACACCTTGCAACCCAAAGACACTTTCAGGCTCTGGTTGACAGCCGAAGTGCACCCAAACTTTACACCTATCCTCCTCCAGTCAAGCCTCAAGATCACTTATGAATCACCACCTGGACTCAAGAAGAATCTCATGCGCACATATGAATCTTGGACACCTGAACAAATCTCTAAGAAAGATAACACTCACCGCGCACATGCTCTGTTCTCTCTGGCCTGGTTTCACGCCGCATGCCAAGAGAGGCGCAACTACATTCCTCAAGGTTGGACCAAATTCTACGAGTTCTCTCTCTCCGACCTGAGGGCCGGTTACAATATCATTGACAGGCTCTTTGATGGTGCCAAGGACGTGCAATGGGAGTTTGTCCATGGTCTGCTGGAGAACGCCATCTACGGAGGCCGCATCGATAACTATTTCGACTTGCGCGTCCTGCAGAGCTATTTGAAGCAATTCTTCAACTCTAGCGTCATTGACGTGTTCAATCAGCGCAACAAGAAGTCCATCTTTCCCTACAGCGTGAGCCTGCCACAAAGCTGCAGCATTCTGGATTACAGGGCTGTCATTGAGAAGATTCCAGAAGACGATAAGCCATCCTTCTTCGGTCTGCCTGCCAACATTGCACGCTCATCACAGCGCATGATCTCATCTCAGGTGATTTCCCAACTCCGCATCCTGGGCCGCTCTATTACTGCAGGCTCAAAGTTCGATCGCGAGATCTGGAGCAATGAACTCAGCCCAGTGCTCAACCTGTGGAAGAAACTGAACCAGAACTCCAACCTGATCCACCAGAAGGTCCCTCCTCCCAATGACCGCCAAGGATCACCAATTCTGTCCTTTATTATCTTGGAGCAGTTCAACGCCATCAGATTGGTCCAGTCAGTTCATCAATCACTGGCAGCCTTGAGCAAAGTGATCCGCGGCACTACACTGCTCTCATCAGAAGTCCAGAAGTTGGCCTCTGCCCTGCTCAACCAGAAGTGCCCTCTGGCCTGGCAATCCAAGTGGGAGGGACCCGAAGACCCTCTGCAATATCTCAGAGGCCTCGTGGCTAGAGCACTGGCCATCCAGAATTGGGTCGATAAAGCAGAGAAGCAGGCCCTCTTGTCCGAAACACTGGACCTCTCTGAATTGTTCCATCCCGACACATTCCTGAACGCCCTCCGCCAGGAAACAGCAAGGGCTGTTGGAAGATCAGTGGATTCTCTGAAATTTGTCGCCTCCTGGAAGGGTAGACTGCAAGAGGCCAAACTCCAGATCAAGATCTCAGGACTGCTCCTGGAAGGCTGCTCCTTCGACGGTAATCAACTGTCCGAGAATCAGCTCGACAGCCCAAGCGTCTCTAGCGTTCTGCCATGCTTCATGGGTTGGATTCCTCAAGACGCTTGCGGCCCATACTCACCCGATGAGTGCATTTCTTTGCCAGTGTACACATCCGCTGAGCGCGACAGAGTCGTGACCAACATCGATGTGCCTTGCGGTGGTAATCAGGATCAATGGATTCAGTGTGGAGCCGCCTTGTTTCTCAAGAATCAGGTCtcgatgccaggcagtccaatgttatgtaaaacgaaggcgaaagggtgtctaacaccatctgatcgatacaaactcgcactggccgcccacaaacgtcatggaaaggtagaaaattcgatgggctgacgctattaccgtatcataggtcgactcacgtgggtacgtgccgtagctcccatgtttaccgcttatgcgggtccagtggaaagagctttactggacgaataacgctgctgctttttaatccatatatgatactgcctagaacaagtacgggaaaatttcgacggcgtgcaatgtgcaatatgtttccgctatttgatcactcttggccgagtgcaatctctcactcgcgcttttgggctaacgacatagactcaatatcttagagtgagacgtgcggtctttcagtggagaaagccctgtttagaccacaggttcctattattcggatgaacgacctttaagataggtcaaccattatgacagttgcctgagtaagaacacgagcggagtatctgattctgatgcttagacgctgtcgcatcccgtgaaagctcatccagaccgggtgagcgtagacctataactacgccaacctacccggccgggaaatcatgtaggcaactcaacccgctcgcatgtaagttgtccataatatgaatttacccatccgaattgtatcgtggagttgttcggctagtggcaggagttcttagactaatgacaccctcactgttgcggcggtaacaaccattaactattacaagttgcggtttggtaaggttagggtaactgtagttaaaagtatttctgcgattgctcgtttcgtcagatcacttcacagcgcagtctacggcactagggacaagatttgtgtactgtggacccgtagccgagaaatccacggcattcatgagacgttactcgggaactattcagtcagtgtatgtagtcggcaaccggtagtggttccggaacaagcttttgaaaatcagttaatgtgggttgagctgctcagaggcgcccagttgtaccggaaaagtatgcatatctttaatggctatgcaagtatccaactatgctggcggcagggtagccatatccatcgggtccagaaggttcccattaaatacccgacactactagctatgttttgggtcctgttcccgagttacctgcataaggtgaataagccttagtaagttactgctctatgacattggctcagtcagactgttgcatacttatcttatagattcctatgcggaaaattccgaccgcagacttaataacttgatctaatgcccggtttctcagtacgatactagttaaaccggccagcacaggttcgtatatatccctgtatggatatgatctcgggtcgactttaagcgactaagtgctctagcgcctgatggcgtgctttcttctcccacgttgctatatcaccgagtgagtacccgcggatagagttgcttcgcaggtactcaaccacagttaggcaagtgcgaaggtattactgattaggcgtggccgcggccgtacacctcgttagtttgagggaagctgttccgatgactggctagcaggcctgggtgagtactagtgatcagcaaagcctctctgtggtcgcataggtccgacaatatgagcgcagtatgcgagtcccaccgaaattttgcaacgatcttgattctccctccagagtctaaatttcctatcctccaagtgtaactccagcatcagatgtttcttagaagtactcggagaatacaactttgagtacaatttgcgcgtccgtccggtttcccggtgggatttaccaacttaaaacttctagaccattattcacagcctagcgctgcgattggcggctcatgtgcggctcggctggtagggttggtgatgtggctaaccgtcgaagcgttgtaggacaccttatttggagaagagacgttgtgacgggaaaagtgtctcatgcgtaatgttccggagtatgggttcctgtgacgtgtagcagccttcaaatggacggatttacgccaatatttcattaagtgagcctaaaattagcccgcaacgaggtgatctgggagactacaatctaaggttgcgagtgtgatcagctcagaaacaatagctcgtgaacctcgattcagcagggattgcactctgtggtccgtttctccactctctggttggtttacgagtgagccctttaaccctggtaggatccataaaacatgtaaaaccaccttgcttgtgctagcattagggaccggtgttcacacccatctatatggaggctcctggtgcacctcggacaaatgaggctttaagctatcgattccgaatggctacctggcgcatgcaaggtgccctgttcatgtccagtcagtgctgatgggaccgctgcgagagacctagtagattcttcgcataaaaatatatgtgttaacacggtactgtccgtggtatgccgttagaaataggtgcccatccgactcacgtcctgccagcgaccaatggaatcccgctaaacagaaatgattatagtgagcaaggcatagtaaatatcagtccggtactactctacttcgagtccctaaagaacgacaaggatctgtgaataaaactatagatcaattcagtgcttaccctcctatctgcttaaggtgtaattgtatgagggtgccgcgaccttcgtcatttcgaaaatgcccagttgacctaaaaccaggcaatgtctagaaccgcatagggacttaatgacaggccgccgatccagcaatgatttattcgccgaatagtcccaagtgttcacgggagatcgttattgggaggcgggtgtatgtgtgagcgccctacacccttctatcttctcgtacggccttgttgagtatttatctgccgacttgatcaaaatagggtccgccatcgtcacattacgacttcgaaatcccgatgtgctgcttagaaattttgtaacatcggtaccaagagagtgcaaaacgaataattgctgagagcagcactgtgcaagactggaaaggtggtgaagcaatacaccatgaatcccggatcgctgacgcgccctgtagcggcgcattaagcgcggcgggtgtggtggttacgcgcagcgtgaccgctacacttgccagcgccctagcgcccgctcctttcgctttcttcccttcctttctcgccacgttcgccggctttccccgtcaagctctaaatcgggggctccctttagggttccgatttagtgctttacggcacctcgaccccaaaaaacttgatttgggtgatggttcacgtagtgggccatcgccctgatagacggtttttcgccctttgacgttggagtccacgttcttt |
| 3031-nt ssDNA sequence for composition test | aatagtggactcttgttccaaactggaacaacactcaaccctatctcgggctattcttttgatttataagggattttgccgatttcggaacgggtacctacgaagagttccagcagggattccaagaaatggccaatgaagattggatcacctttcgcactaagacctacttgtttgaggagtttctgatgaattggcacgaccgcctcaggaaagtggaggagcattctgtgatgactgtcaagctccaatctgaggtggacaaatataagattgttatccctatcctgaagtacgtccgcggagaacacctgtcacccgatcactggctggatctgttccgcttgctgggtctgcctcgcggcacatctctggagaaactgctgttcggtgacctgctgagagttgccgataccatcgtggccaaggctgctgacctgaaagatctgaactcacgcgcccagggtgaagtgaccatccgcgaagcactcagggaactggatttgtggggcgtgggtgctgtgttcacactgatcgactatgaggactcccagagccgcaccatgaagctgatcaaggattggaaggacatcgtcaaccaggtgggcgacaatagatgcctcctgcagtccttgaaggactcaccatactataaaggctttgaagacaaggtcagcatctgggaaaggaaactcgccgaactggacgaatatttgcagaacctcaaccatattcagagaaagtgggtttacctcgaaccaatctttggtcgcggagccctgcccaaagagcagaccagattcaacagggtggatgaagatttccgcagcatcatgacagatatcaagaaggacaatcgcgtcacaaccttgactacccacgcaggcattcgcaactcactgctgaccatcctggaccaattgcagagatgccagcgcagcctcaacgagttcctggaggagaagcgcagcgccttccctcgcttctacttcatcggagacgatgacctgctggagatcttgggccagtcaaccaatccatccgtgattcagtctcacctcaagaagctgtttgctggtatcaactctgtctgtttcgatgagaagtctaagcacattactgcaatgaagtccttggagggagaagttgtgccattcaagaataaggtgcccttgtccaataacgtcgaaacctggctgaacgatctggccctggagatgaagaagaccctggagcagctgctgaaggagtgcgtgacaaccggacgcagctctcagggagctgtggacccttctctgttcccatcacagatcctgtgcttggccgaacagatcaagtttaccgaagatgtggagaacgcaattaaagatcactccctgcaccagattgagacacagctggtgaacaaattggagcagtatactaacatcgacacatcttccgaggacccaggtaacacagagtccggtattctggagctgaaactgaaagcactgattctcgacattatccataacatcgacgtggtcaagcagctgaaccaaatccaagtgcacaccaccgaagattgggcctggaagaagcagttgaggttctacatgaagtccgaccacacctgttgcgttcagatggttgacagcgagttccagtacacctatgagtaccaaggaaatgccagcaagctcgtttacactccactcactgacaagtgttacctcaccttgacacaggctatgaagatgggcctgggaggcaacccatacggtccagctggcactggtaagacagagagcgttaaggcactcggaggtctgctgggcaggcaggtcctcgtgttcaactgtgatgaaggaatcgacgttaagtccatgggaagaatctttgttggcctcgttaagtgtggagcttggggttgcttcgacgagttcaacaggctggaggaatctgtgctgagcgccgtctctatgcagatccagaccatccaggacgcattgaagaaccacaggaccgtctgcgagctgttgggtaaggaagtggaggtgaactccaactccggaatcttcatcacaatgaatcccgcaggtaaaggatatggaggaagacagaaactcccagacaacctgaagcagctgttccgcccagtggctatgtcccatccagacaatgagctgatcgccgaagtcatcctctattccgagggattcaaagatgctaaagttctctccagaaagctcgtggccatcttcaatctgtcaagagaactcctgacacctcagcagcattacgactggggtctgagagccctcaagaccgtcctgagaggttcaggaaatctcctcaggcagctgaacaagagcggtacaacacagaatgcaaatgagagccacattgtcgtccaggctctgaggctgaataccatgtcaaagttcacattcacagactgcacaagatttgacgctctgattaaagatgtgttccctggtattgaactcaaagaagtggagtatgacgagctgagcgccgctttgaagcaggtgtttgaggaggctaactatgagattatccctaatcagatcaagaaagcattggaactgtatgaacagctgtgtcagaggatgggagtggtgattgtgggcccatcaggcgcaggtaagagcactctctggagaatgctgagagcagcactgtgcaagactggaaaggtggtgaagcaatacaccatgaatcccggatcgctgacgcgccctgtagcggcgcattaagcgcggcgggtgtggtggttacgcgcagcgtgaccgctacacttgccagcgccctagcgcccgctcctttcgctttcttcccttcctttctcgccacgttcgccggctttccccgtcaagctctaaatcgggggctccctttagggttccgatttagtgctttacggcacctcgaccccaaaaaacttgatttgggtgatggttcacgtagtgggccatcgccctgatagacggtttttcgccctttgacgttggagtccacgttcttt |
| 3384-nt BCMA CAR template | aatagtggactcttgttccaaactggaacaacactcaaccctatctcgggctattcttttgatttataagggattttgccgatttcggaacgggtacctacgaagagttccCTTACATCTAGTTGAGCTGTCACAGAATGTGACGTTGAAGgatgtaaggagctgctgtgacttgctcaaggccttatatcgagtaaacggtagtgctggggcttagacgcaggtgttctgatttatagttcaAAACCTCTATCAATGAGAGAGCAATCTCCTGGTAATGTGATAGATTTCCCAACTTAATGCCAACATACCATAAACCTCCCATTCTGCTAATGCCCAGCCTAAGTTGGGGAGACCACTCCAGATTCCAAGATGTACAGTTTGCTTTGCTGGGCCTTTTTCCCATGCCTGCCTTTACTCTGCCAGAGTTATATTGCTGGGGTTTTGAAGAAGATCCTATTAAATAAAAGAATAAGCAGTATTATTAAGTAGCCCTGCATTTCAGGTTTCCTTGAGTGGCAGGCCAGGCCTGGCCGTGAACGTTCACTGAAATCATGGCCTCTTGGCCAAGATTGATAGCTTGTGCCTGTCCCTGAGTCCCAGTCCATCACGAGCAGCTGGTTTCTAAGATGCTATTTCCCGTATAAAGCATGAGACCGTGACTTGCCAGCCCCACAGAGCCCCGCCCTTGTGCATCACTGGCATCTGGACTCCAGCCTGGGTTGGGGCAAAGAGCGAAATGAGATCATGTCCTAACCCTGgaattggATCCTCTTGTCttACAGATGGATCTGGAGCAACAAACTTCTCACTACTCAAACAAGCAGGTGACGTGGAGGAGAATCCCGGCCCCATGAAATGGAAAGCACTCTTTACCGCCGCAATCCTTCAAGCACAGTTGCCAATTACCGAGGCTgaacagaagcttatctctgaagaggatcttGACATTGTACTGACGCAAAGTCCCCCTAGCTTGGCGATGAGTCTCGGGAAGCGAGCGACGATTAGTTGCCGAGCTTCTGAAAGTGTCACAATCCTTGGCTCCCACCTGATCCATTGGTACCAACAAAAACCTGGGCAGCCCCCGACGCTTCTCATTCAGTTGGCGTCTAACGTGCAAACAGGAGTACCGGCCAGATTTTCAGGCTCAGGCTCTCGCACCGACTTTACTCTGACCATCGACCCTGTTGAGGAGGATGATGTAGCAGTTTACTACTGTCTTCAGAGCAGAACCATTCCTCGCACATTCGGCGGTGGAACGAAGTTGGAAATCAAGGGCTCAACAAGTGGGAGTGGGAAGCCCGGCAGCGGGGAGGGTTCTACTAAAGGCCAAATACAGTTGGTTCAATCCGGGCCTGAACTGAAAAAGCCGGGAGAGACCGTGAAAATTTCTTGCAAGGCTAGCGGGTACACTTTTACGGATTACTCTATTAACTGGGTTAAGAGGGCACCGGGCAAAGGGCTGAAATGGATGGGCTGGATAAACACCGAGACTCGGGAGCCTGCATATGCTTATGATTTCAGAGGTAGATTTGCGTTCTCTTTGGAAACCTCAGCTTCAACGGCCTACTTGCAGATTAATAACTTGAAGTACGAAGACACCGCCACTTACTTCTGCGCTCTCGATTACTCATACGCTATGGATTACTGGGGCCAGGGCACGTCCGTGACCGTGTCCAGCgcaattgaagttatgtatcctcctccttacctagacaatgagaagagcaatggaaccattatccatgtgaaagggaaacacctttgtccaagtcccctatttcccggaccttctaagcccttttgggtgctggtggtggttggtggagtcctggcttgctatagcttgctagtaacagtggcctttattattttctgggtgaggagtaagaggagcaggctcctgcacagtgactacatgaacatgactccccgccgccccgggcccacccgcaagcattaccagccctatgccccaccacgcgacttcgcagcctatcgctccagagtgaagttcagcaggagcgcagacgcccccgcgtaccagcagggccagaaccagctctataacgagctcaatctaggacgaagagaggagtacgatgttttggacaagagacgtggccgggaccctgagatggggggaaagccgagaaggaagaaccctcaggaaggcctgtacaatgaactgcagaaagataagatggcggaggcctacagtgagattgggatgaaaggcgagcgccggaggggcaaggggcacgatggcctttaccagggtctcagtacagccaccaaggacacctacgacgcccttcacatgcaggccctgccccctcgcGGAAGCGGAGCTACTAACTTCAGCCTGCTGAAGCAGGCTGGAGACGTGGAGGAGAACCCTGGACCCaaTATCCAGAACCCTGACCCTGCCGTGTACCAGCTGAGAGACTCTAAATCCAGTGACAAGTCTGTCTGCCTATTCACCGATTTTGATTCTCAAACAAATGTGTCACAAAGTAAGGATTCTGATGTGTATATCACAGACAAAACTGTGCTAGACATGAGGTCTATGGACTTCAAGAGCAACAGTGCTGTGGCCTGGAGCAACAAATCTGACTTTGCATGTGCAAACGCCTTCAACAACAGCATTATTCCAGAAGACACCTTCTTCCCCAGCCCAGGTAAGGGCAGCTTTGGTGCCTTCGCAGGCTGTTTCCTTGCTTCAGGAATGGCCAGGTTCTGCCCAGAGCTCTGGTCAATGATGTCTAAAACTCCTCTGATTGGTGGTCTCGGCCTTATCCATTGCCACCAAAACCCTCTTTTTACTAAGAAACAGTGAGCCTTGTTCTGGCAGTCCAGAGAATGACACGGGAAAAAAGCAGATGAAGAGAAGGTGGCAGGAGAGGGCACGTGGCCCAGCCTCAGTCTCTCCAACTGAGTTCCTGCCTGCCTGCCTTTGCTCAGACTGTTTGCCCCTTACTGCTCTTCTAGGCCTCATTCTAAGCCCCTTCTCCAAGTTGACGTTGAAGCGTTACCTGTTAGGTAACGTAGTTGAGCTGTCAACTTGGccatgaatcccggatcgctgacgcgccctgtagcggcgcattaagcgcggcgggtgtggtggttacgcgcagcgtgaccgctacacttgccagcgccctagcgcccgctcctttcgctttcttcccttcctttctcgccacgttcgccggctttccccgtcaagctctaaatcgggggctccctttagggttccgatttagtgctttacggcacctcgaccccaaaaaacttgatttgggtgatggttcacgtagtgggccatcgccctgatagacggtttttcgccctttgacgttggagtccacgttcttt |
| 3396-nt BCMA CAR template | aatagtggactcttgttccaaactggaacaacactcaaccctatctcgggctattcttttgatttataagggattttgccgatttcggaacgggtacctacgaagagttccCTTACATCTAGTTGAGCTGTCACAGAATGTGACGTTGAAGGATGTAAGGAGCTGCTGTGACTTGCTCAAGGCCTTATATCGAGTAAACGGTAGTGCTGGGGCTTAGACGCAGGTGTTCTGATTTATAGTTCAAAACCTCTATCAATGAGAGAGCAATCTCCTGGTAATGTGATAGATTTCCCAACTTAATGCCAACATACCATAAACCTCCCATTCTGCTAATGCCCAGCCTAAGTTGGGGAGACCACTCCAGATTCCAAGATGTACAGTTTGCTTTGCTGGGCCTTTTTCCCATGCCTGCCTTTACTCTGCCAGAGTTATATTGCTGGGGTTTTGAAGAAGATCCTATTAAATAAAAGAATAAGCAGTATTATTAAGTAGCCCTGCATTTCAGGTTTCCTTGAGTGGCAGGCCAGGCCTGGCCGTGAACGTTCACTGAAATCATGGCCTCTTGGCCAAGATTGATAGCTTGTGCCTGTCCCTGAGTCCCAGTCCATCACGAGCAGCTGGTTTCTAAGATGCTATTTCCCGTATAAAGCATGAGACCGTGACTTGCCAGCCCCACAGAGCCCCGCCCTTGTCCATCACTGGCATCTGGACTCCAGCCTGGGTTGGGGCAAAGAGGGAAATGAGATCATGTCCTAACCCTGgaattggATCCTCTTGTCttACAGATGGATCTGGAGCAACAAACTTCTCACTACTCAAACAAGCAGGTGACGTGGAGGAGAATCCCGGCCCcatggcacttccagtaactgcgctgctgctcccgctcgcactcctgctgcatgcggcccgaccagaacagaagcttatctctgaagaggatcttGACATTGTACTGACGCAAAGTCCCCCTAGCTTGGCGATGAGTCTCGGGAAGCGAGCGACGATTAGTTGCCGAGCTTCTGAAAGTGTCACAATCCTTGGCTCCCACCTGATCCATTGGTACCAACAAAAACCTGGGCAGCCCCCGACGCTTCTCATTCAGTTGGCGTCTAACGTGCAAACAGGAGTACCGGCCAGATTTTCAGGCTCAGGCTCTCGCACCGACTTTACTCTGACCATCGACCCTGTTGAGGAGGATGATGTAGCAGTTTACTACTGTCTTCAGAGCAGAACCATTCCTCGCACATTCGGCGGTGGAACGAAGTTGGAAATCAAGGGCTCAACAAGTGGGAGTGGGAAGCCCGGCAGCGGGGAGGGTTCTACTAAAGGCCAAATACAGTTGGTTCAATCCGGGCCTGAACTGAAAAAGCCGGGAGAGACCGTGAAAATTTCTTGCAAGGCTAGCGGGTACACTTTTACGGATTACTCTATTAACTGGGTTAAGAGGGCACCGGGCAAAGGGCTGAAATGGATGGGCTGGATAAACACCGAGACTCGGGAGCCTGCATATGCTTATGATTTCAGAGGTAGATTTGCGTTCTCTTTGGAAACCTCAGCTTCAACGGCCTACTTGCAGATTAATAACTTGAAGTACGAAGACACCGCCACTTACTTCTGCGCTCTCGATTACTCATACGCTATGGATTACTGGGGCCAGGGCACGTCCGTGACCGTGTCCAGCgcaaccacgacgccagcgccgcgaccaccaacaccggcgcccaccatcgcgtcgcagccTctgtccctgcgcccagaggcgtgccgAccagcggcgggTggAgcagtgcacacgagggggctggacttcgcctgtgatatctacatctgggcgcccttggccgggacttgtggggtccttctcctgtcactggttatcaccctttaCTGCAAGCGGGGCAGAAAGAAGCTGCTGTACATCTTCAAGCAGCCCTTCATGCGGCCCGTGCAGACCACCCAGGAAGAGGACGGCTGCTCCTGCAGATTCCCCGAGGAAGAAGAAGGCGGCTGCGAGCTGagagtgaagttcagcaggagcgcagacgcccccgcgtaccagcagggccagaaccagctctataacgagctcaatctaggacgaagagaggagtacgatgttttggacaagagGcgtggccgggaccctgagatggggggaaagccgagaaggaagaaccctcaggaaggcctgtacaatgaactgcagaaagataagatggcggaggcctacagtgagattgggatgaaaggcgagcgccggaggggcaaggggcacgatggcctttaccagggtctcagtacagccaccaaggacacctacgacgcccttcacatgcaggccctgccccctcgcGGAAGCGGAGCTACTAACTTCAGCCTGCTGAAGCAGGCTGGAGACGTGGAGGAGAACCCTGGACCCaaTATCCAGAACCCTGACCCTGCCGTGTACCAGCTGAGAGACTCTAAATCCAGTGACAAGTCTGTCTGCCTATTCACCGATTTTGATTCTCAAACAAATGTGTCACAAAGTAAGGATTCTGATGTGTATATCACAGACAAAACTGTGCTAGACATGAGGTCTATGGACTTCAAGAGCAACAGTGCTGTGGCCTGGAGCAACAAATCTGACTTTGCATGTGCAAACGCCTTCAACAACAGCATTATTCCAGAAGACACCTTCTTCCCCAGCCCAGGTAAGGGCAGCTTTGGTGCCTTCGCAGGCTGTTTCCTTGCTTCAGGAATGGCCAGGTTCTGCCCAGAGCTCTGGTCAATGATGTCTAAAACTCCTCTGATTGGTGGTCTCGGCCTTATCCATTGCCACCAAAACCCTCTTTTTACTAAGAAACAGTGAGCCTTGTTCTGGCAGTCCAGAGAATGACACGGGAAAAAAGCAGATGAAGAGAAGGTGGCAGGAGAGGGCACGTGGCCCAGCCTCAGTCTCTCCAACTGAGTTCCTGCCTGCCTGCCTTTGCTCAGACTGTTTGCCCCTTACTGCTCTTCTAGGCCTCATTCTAAGCCCCTTCTCCAAGTTGACGTTGAAGCGTTACCTGTTAGGTAACGTAGTTGAGCTGTCAACTTGGccatgaatcccggatcgctgacgcgccctgtagcggcgcattaagcgcggcgggtgtggtggttacgcgcagcgtgaccgctacacttgccagcgccctagcgcccgctcctttcgctttcttcccttcctttctcgccacgttcgccggctttccccgtcaagctctaaatcgggggctccctttagggttccgatttagtgctttacggcacctcgaccccaaaaaacttgatttgggtgatggttcacgtagtgggccatcgccctgatagacggtttttcgccctttgacgttggagtccacgttcttt |
| 3024-nt scaffold for DNA origami tile | aatagtggactcttgttccaaactggaacaacactcaaccctatctcgggctattcttttgatttataagggattttgccgatttcggaacggattactctacttcctcaacaaaccacattaccatcatcttccatcacttatcaaagtctttatttatccactcctctaccttactaacctcttctatccatataactctctggcttaaatacctcctatacaaattactcctacctctaataattaaaaaatttattgcacacaaataacctctcctaaactcaataatcaaccttacctcctcaccaaataacgaactcctaactactatttcaaaaaacctcttacactcaatatctttaaataaatagtactattcaaacatctccaactacttcacttattacacaattcctcaacactcctaattcaaattacaaacacaccaattacgtaccttatcctttatcaaatttctcggaaactaccacctctcaaccttcatacattaccaccaatacttcattaatatatatcgttattccattatttttaaacaaaccgacaggattggaaggacatcgtcaaccaggtgggcgacaatagatgcctcctgcagtccttgaaggactcaccatactataaaggctttgaagacaaggtcagcatctgggaaaggaaactcgccgaactggacgaatatttgcagaacctcaaccatattcagagaaagtgggtttacctcgaaccaatctttggtcgcggagccctgcccaaagagcagaccagattcaacagggtggatgaagatttccgcagcatcatgacagatatcaagaaggacaatcgcgtcacaaccttgactacccacgcaggcattcgcaactcactgctgaccatcctggaccaattgcagagatgccagcgcagcctcaacgagttcctggaggagaagcgcagcgccttccctcgcttctacttcatcggagacgatgacctgctggagatcttgggccagtcaaccaatccatccgtgattcagtctcacctcaagaagctgtttgctggtatcaactctgtctgtttcgatgagaagtctaagcacattactgcaatgaagtccttggagggagaagttgtgccattcaagaataaggtgcccttgtccaataacgtcgaaacctggctgaacgatctggccctggagatgaagaagaccctggagcagctgctgaaggagtgcgtgacaaccggacgcagctctcagggagctgtggacccttctctgttcccatcacagatcctgtgcttggccgaacagatcaagtttaccgaagatgtggagaacgcaattaaagatcactccctgcaccagattgagacacagctggtgaacaaattggagcagtatactaacatcgacacatcttccgaggacccaggtaacacagagtccggtattctggagctgaaactgaaagcactgattctcgacattatccataacatcgacgtggtcaagcagctgaaccaaatccaagtgcacaccaccgaagattgggcctggaagaagcagttgaggttctacatgaagtccgaccacacctgttgcgttcagatggttgacagcgagttccagtacacctatgagtaccaaggaaatgccagcaagctcgtttacactccactcactgacaagtgttacctcaccttgacacaggctatgaagatgggcctgggaggcaacccatacggtccagctggcactggtaagacagagagcgttaaggcactcggaggtctgctgggcaggcaggtcctcgtgttcaactgtgatgaaggaatcgacgttaagtccatgggaagaatctttgttggcctcgttaagtgtggagcttggggttgcttcgacgagttcaacaggctggaggaatctgtgctgagcgccgtctctatgcagatccagaccatccaggacgcattgaagaaccacaggaccgtctgcgagctgttgggtaaggaagtggaggtgaactccaactccggaatcttcatcacaatgaatcccgcaggtaaaggatatggaggaagacagaaactcccagacaacctgaagcagctgttccgcccagtggctatgtcccatccagacaatgagctgatcgccgaagtcatcctctattccgagggattcaaagatgctaaagttctctccagaaagctcgtggccatcttcaatctgtcaagagaactcctgacacctcagcagcattacgactggggtctgagagccctcaagaccgtcctgagaggttcaggaaatctcctcaggcagctgaacaagagcggtacaacacagaatgcaaatgagagccacattgtcgtccaggctctgaggctgaataccatgtcaaagttcacattcacagactgcacaagatttgacgctctgattaaagatgtgttccctggtattgaactcaaagaagtggagtatgacgagctgagcgccgctttgaagcaggtgtttgaggaggctaactatgagattatccctaatcagatcaagaaagcattggaactgtatgaacagctgtgtcagaggatgggagtggtgattgtgggcccatcaggcgcaggtaagagcactctctggagaatgctgagagcagcactgtgcaagactggaaaggtggtgaagcaatacaccatgaatcccggatcgctgacgcgccctgtagcggcgcattaagcgcggcgggtgtggtggttacgcgcagcgtgaccgctacacttgccagcgccctagcgcccgctcctttcgctttcttcccttcctttctcgccacgttcgccggctttccccgtcaagctctaaatcgggggctccctttagggttccgatttagtgctttacggcacctcgaccccaaaaaacttgatttgggtgatggttcacgtagtgggccatcgccctgatagacggtttttcgccctttgacgttggagtccacgttcttt |
| Staple for DNA origami tile | GGCATCTATTGTCGCCCACCTTCTGGTCAAAATGTATGGTGGGAGCCCCTTGCTTC |
| Staple for DNA origami tile | TGCTCTTGTCATGATGCTGCGCCCAAGAACAGTAAAGGTTCACCTTTCCCTCAGCT |
| Staple for DNA origami tile | AGGTTTTGGGGTCGAGGGAGTGTTGTTCCAGTTTGGAATCAAGTTTAAACCC |
| Staple for DNA origami tile | AATAAACCATCGGAAGAAAGCGAAACCACACCGGAACTCTTGCAGTAATGTGCCGTTATT |
| Staple for DNA origami tile | TGTGACGCGATTGTCCTTCTTTCGTCTCGGATTCACACAGTGCTGCTCTAACACCT |
| Staple for DNA origami tile | TGGGTAGTCAAGGTCGAGGGAACAGGGCGCGTCAG |
| Staple for DNA origami tile | TCTCCAGTGAGGTGAGACTGACCTTCAGCGGGAGTAGGTATCATACTCCCTCTCAG |
| Staple for DNA origami tile | GGCACCTGACTTCAGTTGAGGGGTCAGCGGTTCTGCCCAGATGCTGACCTTGTCTTCTG |
| Staple for DNA origami tile | CAGCTGCCACAGCTCCCTGAGAATACCGGATATTGTTGATCGGTCTTGA |
| Staple for DNA origami tile | CATCTCCAGGGCCAACAGAGTTGATACCAGCAAACTCTTCTTGCTTCAATGAGTTCAATA |
| Staple for DNA origami tile | GTTTCGATTAGACTGCGCTTCGCCTGCGACTTTCTCAAAGCCTTTATAGTATGGTGTTCG |
| Staple for DNA origami tile | GACTCTGATAATGTCGAGAATGGAGTGT |
| Staple for DNA origami tile | ACCAGCTGCCCAATCTTCGGTACCATCTACTTCGGATTCCGGAGTTGGAAATGCGT |
| Staple for DNA origami tile | GTTATGGTGTTACCAAGGGTCTCCAGGGAGCTTCTCAGGTCAGATATCTTGGGCAG |
| Staple for DNA origami tile | CAGCTGCTTGACCACGTCGATGCATTTC |
| Staple for DNA origami tile | CTCCCAGGCCCACTTAACATTCCTCCAGCCTGTTTCTGTACAGCTGGGGCTCTCCTGAAC |
| Staple for DNA origami tile | ATTGTGTAATAAGTGTGAGGTAGTGAGTCAGTGCTGCTCCAGAGCTGCGACGCACT |
| Staple for DNA origami tile | TTCTTCCCATGGACTTAACGTTTACCAGGCTGTCAGGTGTGCGCTCCAATGATCTG |
| Staple for DNA origami tile | CGATTCCCCTGGATGGTCTGGGATGAAGCGATCAGGTTCTCTTTCTGTGGCGTCAA |
| Staple for DNA origami tile | TTCATCACAGTTGAAGTGCCTACAGGTGCTTCCAGGTGTCTCTCTCCAC |
| Staple for DNA origami tile | TCCTGTGGTTCTTCGTTCACCCGGAATATGGCCACACAATGTGTGAATGCAATGCTCACA |
| Staple for DNA origami tile | ATCTGCATAGAGACGGCGCTCTTTACCTGCGGGATTCATTGT |
| Staple for DNA origami tile | ATTGAGTTTTTGAAGGTAAGGAGTTTAGTTATTAGAATTTGTATAGAAGGGTAGAG |
| Staple for DNA origami tile | TTAGGTTGTAAGGTCTGGGAGTTGAACTACCCCAAGCTCCACATCTTCACTTGCTG |
| Staple for DNA origami tile | CTTCAGGAAACGAGTAGCCTGTGTCAAGGAAAGCACGTCGAGTAGTTGG |
| Staple for DNA origami tile | GGGCGGACTTCCTCCATATCCAGCACAGGAGGCCAACAAAGATATGGGTTGC |
| Staple for DNA origami tile | AATGCTGCTGAGGTGTCAGGACTCATTGGGAACTCTGCCAGCTGGACCG |
| Staple for DNA origami tile | TGACAGATTGAAGAGAGGATGGAACGCATAACGCTCTCTGTC |
| Staple for DNA origami tile | GAGATTTCAGACCCCAGTCGTAGCCACTCTTGGTACTCATAG |
| Staple for DNA origami tile | ACTTCTTAGCGGCGAGTCTTGTGGTGTACGATTTAAAATCGGAGAATAGCCCGAGATAGG |
| Staple for DNA origami tile | CCAGCCTGAGTCGGAAGATGTGTCGGATCTGTGATGGGAACAGAGTGGGTCC |
| Staple for DNA origami tile | TTTAATCAGATTGTACCGTATACTACTTGGATTTGGTTGTGTACTTCTGGATGGGACAT |
| Staple for DNA origami tile | TTCTTGAGGACAAGATCTTCGGTAAACTTTTGTTCATTTGCA |
| Staple for DNA origami tile | AGTTAGCCTCCTCACAGCATTCTCCAGAGAGTGCT |
| Staple for DNA origami tile | ATCTCATCAGCCAGTTCGGCCAAGCACAGATGTTAGCTCTTGTTCAGCTGGGAACACATC |
| Staple for DNA origami tile | AGGGATACTTACCTCTTAATGAAGGAAGACCCAAACAAGAGTCCACTATTAAAGAA |
| Staple for DNA origami tile | TCTGATTATCTTGTGCAGTCTGGCTCTC |
| Staple for DNA origami tile | GAAGATGGGTTTGTCAAAATCCCTTATAAATCAAAAACCCTA |
| Staple for DNA origami tile | AAGCCGGCGAACGTGGCGAGACGCCGCTAGGCGCTTCTCATCGAAACAGGATCGTT |
| Staple for DNA origami tile | CTACGTGTGGTTGAAGTGAGTTGCGAATTCCTCCACGCCGCGGCGCCTGATGGGCC |
| Staple for DNA origami tile | AAGCACTGAGCTTGACGGGGACGATCCGCGATGAAGTAGAAG |
| Staple for DNA origami tile | GTTTGCCGTACGACCAAAGATTGGAGTCCTTCAAGGACTGCAGGAGGCTCCG |
| Staple for DNA origami tile | TTTTCGGCGAGTTTCCTTTCAAATATAGGGCGAGCGCTAGGGCGCTGGCAAGTTT |
| Staple for DNA origami tile | TTTGCAATTGGTCCAGGATCTGCGCTGCTGCGCACCACTCCCATCCTCTGACTTT |
| Staple for DNA origami tile | TTTAACTTCTCCCTCCAAGTATTCTTACAGTTCTGAACTTTGACATGGTATTTTT |
| Staple for DNA origami tile | TTTTGATCTTTAATTGCGTAATCTGGCTGGACGGAGCTTTCTGGAGAGAACTTTT |
| Staple for DNA origami tile | TTTAGAACCTCAACTGCTTTGGTCGGAATCCCTTCCACTTCCTTACCCAACATTT |
| Staple for DNA origami tile | TTTGCCCAGCAGACCTCCGACACGAG |
| Staple for DNA origami tile | TTTGCTCGCAGACGGGACCTGCCTTTT |
| Staple for DNA origami tile | TTTTTAGCATCTTTGACTTCATGTTTT |
| Staple for DNA origami tile | TTTCAGCCTCAGAGCTGCAGGGAGTTT |
| Staple for DNA origami tile | TTTACAGCTGTTCATGAATGGCACTTT |
| Staple for DNA origami tile | ATCGTAACCAGGAGCGGTGGCCCACGTGGACTCCAACGTCAAAGGGCGAATTT |
| Staple for DNA origami tile | TTTTGTAGCGGTCACGGCATCTCTTTT |
| Staple for DNA origami tile | TTTAAACCGTCTATCTCGTCCAGTTTT |
| Staple for DNA origami tile | TTTCGGTTTGTTTAAGTAGAGTAATTT |
| Staple for DNA origami tile | TTTATATATATTAATTAAATAAAGTTT |
| Staple for DNA origami tile | ATCACGGTGACTGGGAAATCTTGTTGAAGGTTGACGATGTCCTTCCAATCCTGTTTT |
| Staple for DNA origami tile | TTTTGTATGAAGGTTGTATTTAAGTTT |
| Staple for DNA origami tile | TTTCGAGAAATTTGATATTTGTGTTTT |
| Staple for DNA origami tile | TTTTAATTGGTGTGTTTAGGAGTTTTT |
| Staple for DNA origami tile | TTTGAGTGTTGAGGATGAATAGTATTT |
| Staple for DNA origami tile | TTTCTATTTATTTAAAGATAGATGTT |
| Staple for DNA origami tile | TTTCGTTATTTGGTGAGGAATAGTAGTTTTGTCAACACGTAATTTGAATTAGTTT |
| Staple for DNA origami tile | TTTGCAATAAATTTTTTAAGAGAGGTTATTTCATTCAGAAGGATAAGGTACGTTT |
| Staple for DNA origami tile | TTTCCAGAGAGTTATATGGATAGGAGGATTGTCTCCGGGAGGTGGTAGTTTCTTT |
| Staple for DNA origami tile | TTTACTTTGATAAGTGATGGAGTGGAGATTGGTATGGAAGTATTGGTGGTAATTT |
| Staple for DNA origami tile | TTTTCCGTTCCGAAATCGGTGAGGAAAACACCCTCATCATAATGGAATAACGTTT |
